# Updates on Biogenic Metallic and Metal Oxide Nanoparticles: Therapy, Drug Delivery and Cytotoxicity

**DOI:** 10.3390/pharmaceutics15061650

**Published:** 2023-06-03

**Authors:** Maria P. Nikolova, Payal B. Joshi, Murthy S. Chavali

**Affiliations:** 1 Department of Material Science and Technology, University of Ruse “A. Kanchev”, 8 Studentska Str., 7017 Ruse, Bulgaria; 2 Shefali Research Laboratories, 203/454, Sai Section, Ambernath (East), Mumbai 421501, Maharashtra, India; payalchem2@gmail.com; 3 Office of the Dean (Research), Dr. Vishwanath Karad MIT World Peace University (MIT-WPU), Kothrud, Pune 411038, Maharashtra, India; murthy.chavali@mitwpu.edu.in

**Keywords:** biosynthesis, drug delivery, cytotoxicity, biogenic nanotechnology, metallic and metal oxide nanoparticles

## Abstract

The ambition to combat the issues affecting the environment and human health triggers the development of biosynthesis that incorporates the production of natural compounds by living organisms via eco-friendly nano assembly. Biosynthesized nanoparticles (NPs) have various pharmaceutical applications, such as tumoricidal, anti-inflammatory, antimicrobials, antiviral, etc. When combined, bio-nanotechnology and drug delivery give rise to the development of various pharmaceutics with site-specific biomedical applications. In this review, we have attempted to summarize in brief the types of renewable biological systems used for the biosynthesis of metallic and metal oxide NPs and the vital contribution of biogenic NPs as pharmaceutics and drug carriers simultaneously. The biosystem used for nano assembly further affects the morphology, size, shape, and structure of the produced nanomaterial. The toxicity of the biogenic NPs, because of their pharmacokinetic behavior in vitro and in vivo, is also discussed, together with some recent achievements towards enhanced biocompatibility, bioavailability, and reduced side effects. Because of the large biodiversity, the potential biomedical application of metal NPs produced via natural extracts in biogenic nanomedicine is yet to be explored.

## 1. Introduction

Different prokaryotic and eukaryotic organisms, their primary or secondary metabolites, or biomolecules contain components capable of reducing metal salts into metallic or metallic oxide nanoparticles (NPs). For example, in plants and especially in their leaf extracts, there are plenty of phytochemicals, such as terpenoids, ketones, aldehydes, carboxylic acids, phenols, flavones, etc., which are thought to be involved in the synthesis of metal or metal oxide [1]. Renewable natural resources (such as plant extracts, microorganisms, algae, etc.) are used as biological precursors to synthesize NPs, avoiding the production of toxic and harmful by-products. Eco-friendly biosynthesis is useful for minimizing waste and reduction of pollution by utilizing sustainable and renewable feedstock. In contrast to chemical or physical methods that require highly toxic reductants or high radiation, as well as high temperature and pressure [2], biosynthesis utilizes low energy for the initiation the bio-reduction. Moreover, the implementation and use of toxic chemicals and solvents prevent the use of NPs in a range of biomedical and clinical applications [3].

The biogenic synthesis of NPs includes the adoption of unicellular and multicellular entities that operate as a pattern for the formation, assembly, and organization of nano-scaled materials or the assembly follows a bottom-up production route. Various metallic and metal oxide NPs have been synthesized from biogenic precursors by using different process parameters such as pH, pressure, temperature, solvents, etc. The term “biosynthesis” is usually used to denote the production of substances or compounds from simple precursors by living organisms, their metabolites or biomolecules. Ionic liquids are sometimes used instead of other solvents because they easily dissolve organic compounds, gases, or catalysts even though they have a polarity comparable to alcohol. They also operate in large temperature ranges and their solubility can be modified by ions associated with them [1]. Thus, scientists have utilized tissues/organs or whole organisms as the main bioreactors to execute the biosynthesis of metallic and metal oxide NPs with different properties. The production of NPs arising from the resistance mechanism of a certain living organism against a specific metal may alter the chemical nature of the toxic materials making them less- or non-toxic [4]. The natural synthesis can be a result of the bioreduction of the metal ions by enzymes that are oxidized or linking peptides that assemble into a more stable nanostructure [5]. This means that the well-known instability of NPs in solutions due to agglomeration and dissolution can also be overcome by the natural capping mechanism that usually occurs during biosynthesis. Simultaneously, by using different organisms or extracts, biomolecules, or bioactive compounds in the presence of metal salts, researchers can produce NPs of various shapes, sizes, compositions, and biological activities, such as antimicrobial, anticancer, larvicidal, and antioxidant ones [6]. The strong bounding between biological structures by their functional groups and nanoparticles can produce benign nanoscale materials with demanding sustainable advantages.

**Figure 1:**
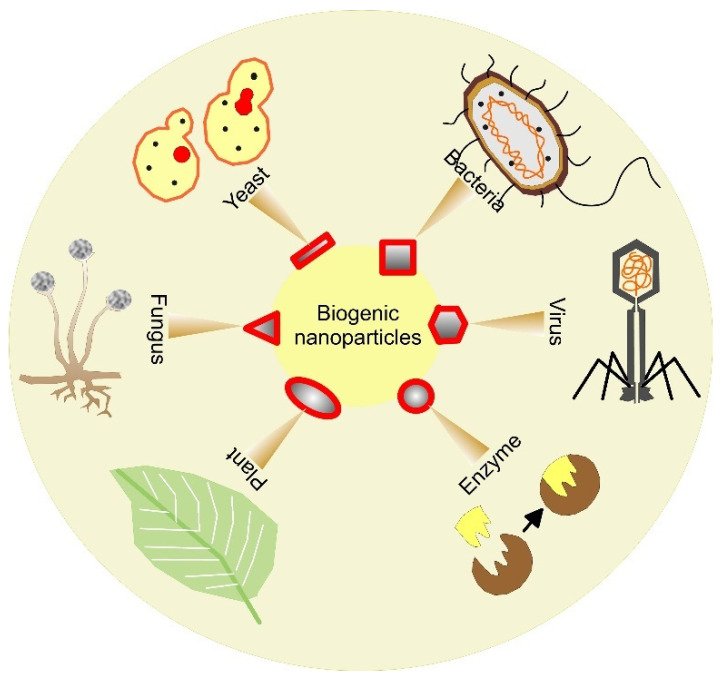
Biosynthesis of biogenic NPs using different species, plant extracts, or macromolecules leading to the formation of structures with various compositions, shapes, and sizes.

Nanotechnology plays a major role in today’s biomedicine. Nanoparticles display distinction over classical therapy methods in terms of effectiveness and competence. Drugs or other active compounds can be loaded in bioengineered NPs for effective transfer to a certain site in a living organism. On the one hand, noble metal NPs such as gold (Au), silver (Ag), palladium (Pd), platinum (Pt), other metals like copper (Cu and its oxide CuO), and some other oxides (zinc oxide, titanium oxide, iron oxides, etc.) are characterized by their outstanding physical-chemical, optical, magnetic, and biological properties [7]. On the other, except in allopathic medicines, worthy pharmaceutical compounds as key sources of antioxidants, antimicrobial, and cytotoxic moieties have been produced by using biomaterials from plants, herbs, different species, and macromolecules (Figure 1) with fewer side effects [1]. Natural materials from renewable energy sources also use safe solvents and reactants and minimize waste products that have health, social, and environmental benefits. Thus, biogenic NPs for biomedical applications have been engineered so that they have improved bioactive performance by complimenting the efficacy of NPs with that of the capping biological agent or are specifically directed towards the diseased cells while decreasing the side effects. A considerable advance has been achieved in many biogenic nanoengineering processes for biomaterial production. The commercialization of successful cost-effective and eco-friendly technologies is of utmost importance for humans but a critical view is necessary for the potential side effects and toxicity of these materials. For that reason, in this review, we have attempted to summarize the biogenic principles of bio-assembly and the construction of nanosystems with biomedical applications. Since many nano drugs rely on metal and metal oxide NPs, we have focused on the biosynthesis of these nanomaterials for therapeutics by giving a background on bio-nanotechnology prospective evolution. Furthermore, some significant applications of biogenic NPs such as cancer therapy and the delivery of antitumor drugs, antimicrobial/antifungal therapy, anti-inflammatory, wound healing, osteoinduction, anti-viral, and antiparasitic functions, as well as their nanotoxicity, have also been discussed.

## 2. Characteristics of Some Biosynthesized NPs

Nanoparticles have been known to be utilized for a great number of biomedical and pharmaceutical applications. When NPs are used, there is a huge increase in surface area available for reactions and hence an increased effect is observed. Nanomaterials have not only a large specific surface area but also a high surface area to volume ratio which increase with the decrease in size, morphology, and distribution of NPs [7]. Also, the electronic configurations of metal NPs are such that they allow the acceptation or donation of an electron to quench free radicals [8]. Metallic NPs can adopt various physical geometries and electronic assemblies that allow them to exhibit metallic, semiconducting, or insulating characteristics.

NPs have been used in various biological and pharmaceutical applications because of their small size (1–100 nm) and similarity to cellular components that allow them to enter living cells by using numerous cellular mechanisms. Nanoparticles provide great advantages in the pharmaceutical industry as drug carriers due to their protection of drugs in vivo, increased and sustained drug activity, improved delivery efficiency and selectivity, and extended release profile [9]. The nano size of the particles also enlarges the penetration potential thus aiding in better utilization of NP properties. Thus, because of the higher site-specific delivery of drugs, the required dose of drugs and side effects will be reduced substantially. In nanosized form, NPs can penetrate the circulatory system and translocate the blood-brain barrier. Such NPs interact in different ways with a large range of biomolecules thus directing various cellular, physicochemical, and biochemical properties [10]. Most biological entities assist stabilization and act as capping agents protecting from coalescence and aggregation. Agglomeration is a phenomenon where the NPs lower the surface energy resulting in a reduction in surface area by increasing the particle size. The biopolymers enhance the biocompatibility of these NPs and prevent agglomeration in clusters that affect their bio-dispersity [11]. The stability of a NP can modulate the biological response by changing the bioavailability, cellular uptake, and toxicity in vitro and in vivo [11]. Factors that manipulate the stability of NPs are based on composition, surface properties, and aggregation state. The combination of molecules from a biological origin that participate in the reduction and capping of NPs promotes their stability, inhibits agglomeration, and impacts their therapeutic effect [7].

Historically, the process of bio-nano-material fabrication dates back to 1980, when Beveridge et al. synthesized gold NPs by using *Bacillus subtilis* [12]. Today, the most commonly examined metallic NPs are Au, Ag, Pt, Pd, Cu, ZnO, and TiO_2_ [13].

### 2.1. Gold NPs

Au NPs are unique with enhanced active properties, unlike their bulk noble counterparts. Au NPs were extensively used in cancer therapy, drug delivery, imaging, and other biomedical applications such as antibacterial [14,15]. They demonstrate outstanding surface plasmon resonance and can be combined with different biological assemblies, such as oligosaccharides, and proteins to enhance their functions. In the typical chemical synthesis of gold nanoparticles, chloroauric acid is reduced using NaBH_4_ or sodium citrate as reducing agents, and, at times, seeded growth control is performed to maintain their uniform nanoparticle morphologies. With the use of Au NPs in medicine, these chemical protocols seem detrimental and toxic to humans. Numerous efforts are taken in preparing hybrid AuNPs through chemical processes that involve drug–gold complexation and require surfactants and reducing agents. Various organisms like plants (*Lonicara japonica*) [16], bacteria (*Delftia acidovorance*) [17], fungi (*Aspergillum* sp.) [18], algae [19], etc. are used for the biosynthesis of Au NPs. The production is usually initiated by tetrachloroaurate salt (HAuCl_4_) while the most common shape is spherical, hexagons, and triangles. The preparation of hybrid AuNPs utilizing biomolecules such as proteins [20] and antibodies [21] is experimented with and has shown efficacy in cancer therapy. Choosing the right materials for biogenesis with a high abundance of reactive compounds, the reduction and capping properties of biocomponents assure stability for different biomedical applications.

### 2.2. Silver NPs

Silver NPs are massively used in many biomedical applications. Ag NPs have been widely utilized in pharmaceutics, medical implant coatings, and wound dressing because of their antimicrobial, anti-inflammatory, and antioxidant properties [22,23]. Silver was found to be the most frequently used nanomaterial (435 products) [24]. Ag NPs are characterized by high sensitivity, conductivity, and chemical stability. Ag NPs exhibit antitumor activity by reducing cell proliferation and promoting intracellular ROS, DNA damage, and apoptosis [25]. It is also well known that silver is highly toxic to microorganisms, including 16 major species of bacteria [26]. Nonetheless, some microorganisms may survive and grow under certain metal ion concentrations due to their resistance to silver. It is thought that the main mechanisms triggering resistance involve the presence of nitrate reductase enzyme [27]. The synthesis of Ag NPs through plant extracts (*Madhuca longifolia*) [28], bacteria (*Bacillus subtilis*) [29], fungi (*Beauveria bassiana*) [30], and algae (*Botryococcus braunii*) [31] with different sizes and shapes have been reported. AgNO_3_ is usually used as precursor salt for biomimetic synthesis while the obtained NPs commonly have spherical, triangular, or hexagonal shapes. The biosynthesis of Ag NPs enhances their stability and may reduce their toxicity. However, despite many therapeutical and medical benefits, depending on the size, shape, and capping agents there may be a problem with the nanotoxicity of silver NPs in humans during long exposition time which will be discussed later in this study.

### 2.3. Platinum and Palladium NPs

Platinum (Pt) and palladium (Pd) NPs have exhibited potential therapeutic effects apart from their excellent catalytic prowess in chemical reactions. Both NPs were exploited in antibacterial and biomedical applications [32]. Few studies revealed that Pt NPs can be biosynthesized from cyanobacteria [33], seaweeds [34], tea extracts [35], honey [36], and eggs [37]. Platinum salts such as H_2_PtCl_6_, K_2_PtCl_6_, K_2_PtCl_4_, PtCl_2_, Pt(AcAc)_2_, Pt(NH_3_)_4_(OH)_2_, Pt(NH_3_)_4_(NO_3_)_2_, and Pt(NH_3_)_4_Cl_2_ are applied for biosynthesis [38]. Although the biomimetic synthesis of Pt NPs is limited, a concise review to understand its mechanism and factors that influence NP morphologies has been conducted [39].

Among nanoparticles for biomedical use, Pd is among the least examined. Pd is a high-density metal. Pd NPs have significant thermal and chemical stability and can be biofunctionalized to become suitable for biomedical applications. It also possesses antimicrobial, antioxidant and cytotoxic activity [40]. The biosynthesis of palladium (Pd) NPs is majorly reported from plant-based extracts such as leaf extracts [41], peel extracts [42], bark extracts [43], fruit extracts [44], root extracts [45] and plant gums [46]. For biosynthesis, K_2_PdCl_4_ solution is usually used. Natural antioxidants, such as monosaccharides, vitamin C, and gallic acid, have been used as reducing agents in Pd NP biosynthesis [47].

### 2.4. Copper and Copper Oxide NPs

Cu NPs are widely utilized as cheap and effective bactericidal agents largely applied in medical treatments [48]. Nowadays, as a promising contender at lower cost, Cu NPs are taking the place of Au and Ag NPs but Cu NPs are highly oxidant in air and water. Another challenging job is to stabilize the NPs after synthesis. The main advantage of using a biogenic route for the production of Cu NPs is stabilization [49]. For example, capping agents in the phytosynthesis of Cu NPs help them to stabilize for more than 30 days in contrast to chemically produced ones that settle down after 24 h [50]. Biosynthesis involves the use of algae [51], sea cucumber [52], microorganisms [53], and plants [54]. According to Ying et al., since copper is a transition metal, usually Cu NPs cannot be directly obtained from simple copper salt [55], while in biosynthesis cupric acetate (monohydrate (CH_3_COO)_2_Cu.H_2_O) [56] or CuSO_4_ [49] are often used for the direct bio-production.

After oxidation, copper oxide (CuO) can be formed. It is a p-type semiconductor compound with a monoclinic structure. CuO NPs are characterized by their antimicrobial properties and ability to easily cross biological barriers to reach target organs according to their size and surface properties [57]. Biosynthesized CuO NPs have been prepared from leaf extract of *Eucaliptus globulos* [58], mint leaves and orange peels [59], algae (*Anabaena cylidrica*) [60], and bacteria (*Serratia* sp.) [61], usually by using copper sulfate salt precursor. The biosynthesized CuO NPs are thought to have increased antimicrobial activity compared to commercial NPs due to the participation of natural extracts used for reduction [3]. However, CuO NPs were found to be highly toxic compared to other metal oxide nanomaterials [62].

### 2.5. Zinc Oxide NPs

ZnO NPs are multifunctional n-type semiconductors with high selectivity and low toxicity, making them suitable for drug delivery and targeted therapy [63]. ZnO NPs are considered one of the most promising antibacterial agents because of their low cytotoxicity, compatibility, and good heat resistance [63]. Chemically, the surface of ZnO NPs is rich in -OH groups allowing ZnO to dissolve slowly in both acidic (as in tumor cell microenvironment) and strong basic conditions. For that reason, in medicine, it is used as an antiplatelet agent, anti-inflammatory, anti-angiogenesis, gene- and drug delivery, dental material, and anti-cancer agent [64]. Due to its good antimicrobial and disinfectant properties, ZnO has found widespread utilization in various dermatological substances [65]. However, the application of ZnO NPs can be limited because of the wide energy bind gap (3.37 eV) and the high complexation of photogenerated electron-hole pairs [66]. Fortunately, doping with other metal ions such as Au, Ag, Pd, etc., or compounding/capping with other non-metal materials such as chitosan [67] can reduce the wide bind gap and strengthen the antibacterial effect. For its biosynthesis, plants (*Mentha pulegium*) [68], bacteria (*S. aureus*) [69], fungi (*Xylaria acuta*) [70], and algae (*Sargassum multicum*) [71] have been used. The precursor salt solution can be zinc nitrate (Zn(NO_3_)_2_·2H_2_O) [67] or zinc acetate dihydrate (Zn(C_2_H_3_O_2_)_2_·2H_2_O) [72].

### 2.6. Other Metal Oxide NPs

Iron oxide NPs are commonly used in target drug delivery, MRI, diagnosis of cancer, and tissue engineering [73]. As a result of Neelian and Browning relaxations, magnetite (Fe_2_O_3_) and maghemite (γ-Fe_2_O_3_) NPs respond to an external magnetic field and can be naturally directed toward magnetic targeting to remotely control the distribution of drug molecules. As magnetic material, iron oxide NPs exposed to alternating magnetic fields can convert electromagnetic energy into heat, which makes them suitable agents for hyperthermia therapy of cancer. The magnetic properties of iron NPs can be tuned by changing the size and shape of the nanocrystals, inducing core-shell coupling, doping with other metals, or forming nanoclusters by crosslinking or encapsulation [74]. The biosynthesis of Fe_3_O_4_ NPs allows the production of particles with a size of 2–80 nm, which is much smaller than the 87–400 nm size of the same wet chemically synthesized Fe_3_O_4_ particles [75].

The three main polymorphs of titanium dioxide (TiO_2_) NPs are anatase, rutile, and brookite. Recognition of TiO_2_ NPs for their unique thermal, electric, optic, catalytic, magnetic, and antimicrobial properties for various applications was found in the literature. Titanium dioxide NPs have the potential to induce cell death since they form reactive oxygen species when their aqueous solution is illuminated with visible or UV light [76]. This property utilized for treating cancer is called photodynamic therapy. The biosynthesized TiO_2_ NPs are ecologically friendly and demonstrate high oxidizing potential [77]. When comparing the anticancer activity of plain and biomodified TiO_2_ NPs, the superior performance of the biogenic NPs was also demonstrated [78]. Moreover, plant-mediated production of TiO_2_ NPs resulted in a synthesis of nanomaterial, having functional groups such as tannins and phenols, which help in the stabilization of TiO_2_ NPs and their increased antioxidant potential [79].

Cerium oxide (CeO_2_) NPs have also received much attention because of their improved redox properties as opposed to their bulk counterpart [80]. In the biomedical field, CeO_2_ NPs are mostly used as therapeutic agents, drug-delivery carriers, and antioxidant, antimicrobial, and antiparasitic ointments because of their unique surface properties and their chemistry, biocompatibility, and high stability [81]. The presence of a mixed valence state (both Ce^3+^ and Ce^4+^) and quick transition of the oxidation states play an important role in the scavenging reaction of oxygen while the surface Ce^3+^:Ce^4+^ ratio influences biological interactions [80]. Biogenic synthesis of CeO_2_ NPs has been reported by using plant extracts [82], bacteria [83], fungi [84], algae [85], or biological products [86]. Biogenic CeO_2_ NPs have shown promising results in treating drug-resistant pathogens and fungi, as well as cancers such as colon, cervical, breast, and osteosarcoma ones [81].

## 3. Biogenic Metallic NPs Production

**Figure 2:**
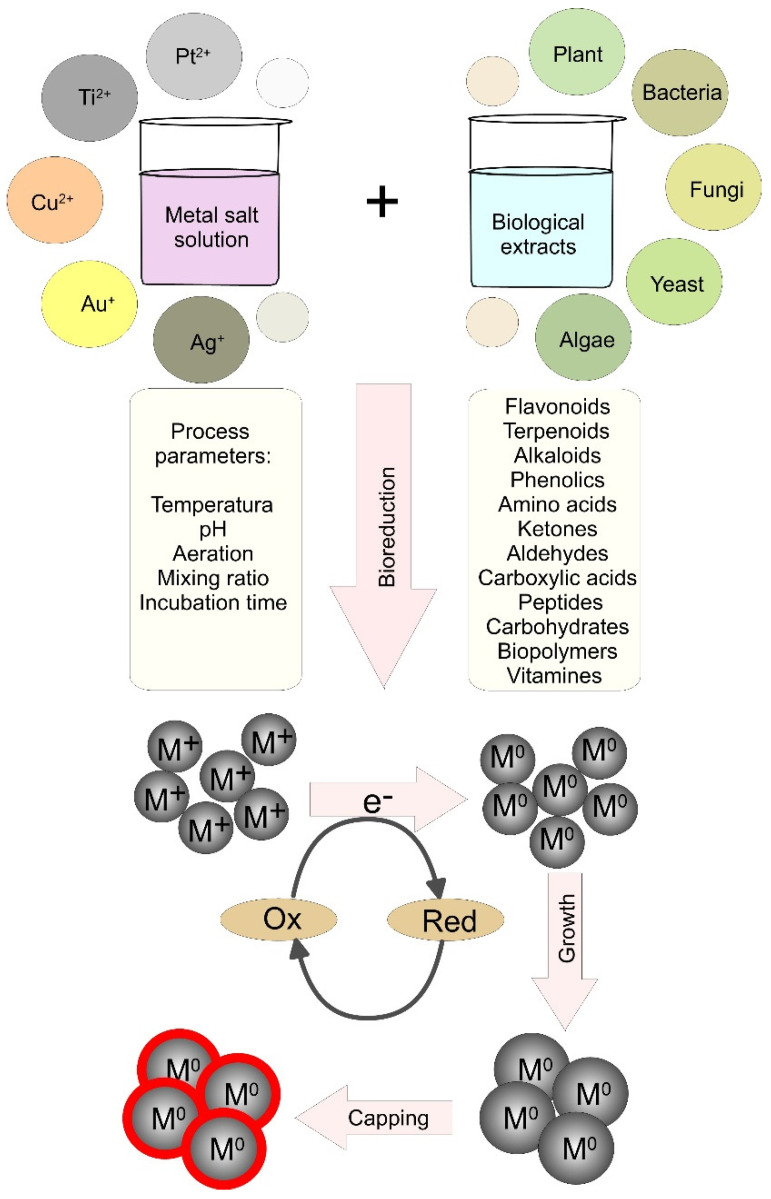
A mechanism, participating components, and factors affecting the biosynthesis of metallic nanoparticles.

To biosynthesize metallic NPs, the living matter uses dissimilatory metal reduction at the expense of the oxidation of enzymes. The different compounds such as terpenoids, flavonoids, alkaloids, glycosides, proteins, carbohydrates, vitamins, polymers, and antioxidants act as reducing and capping/stabilizing agents in the synthesis of sustainable NPs [1]. Figure 2 illustrates the mechanisms, participating components and the affecting factors of the biogenic fabrication of NPs using plants and microorganisms. Despite being sustainable and environmentally friendly, the biosynthesis could be sluggish and time-consuming while the synthesized NPs may be not monodispersed [87]. For that reason, optimization of the control factors and careful selection of the organisms may allow the implementation of such methods in large-scale production. After optimization, the time for biogenic production could be much shorter than physicochemical synthesis. For example, by using brown alga *Padina pavonia*, Abdel-Raouf et al. were able to synthesize silver NPs within 2 min [88]. Adding 200 mg of *P. pavonia* ethanolic or chloroform extract the color changed from colorless to brownish black within 2 min suggesting the formation of silver NPs whose sizes ranged from 49.6 to 86.4 nm. Moreover, to activate the process, a fusion of biogenic methods with alternative routes such as ultrasound and microwave was also applied [89]. Microwave heating, for example, provides fast primary heating and enhanced reaction kinetic thus boosting the reaction rate and increasing the yields [6].

**Figure 3:**
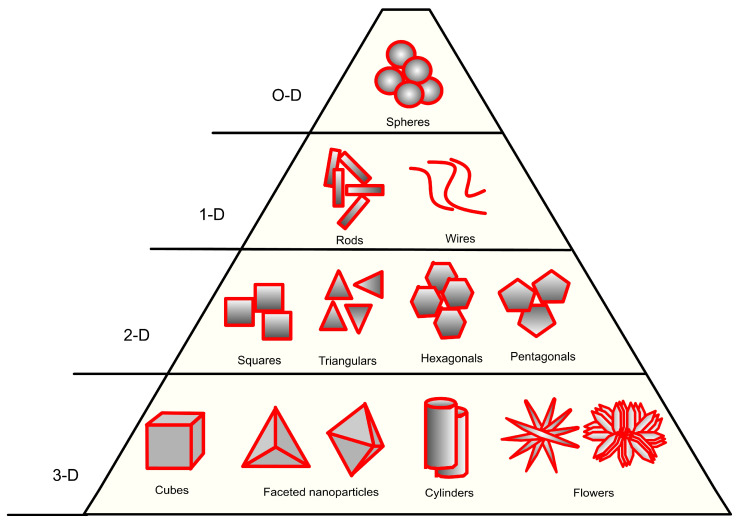
Different shapes of biosynthesized nanoparticles.

The biosynthesis process offers simple and eco-friendly production, and by controlling the process, particles with desired geometries and composition can be formed. Various NPs with different morphology, such as square, rectangular, triangular, polygonal, spherical, cylindrical, flower-like, etc., can be obtained (Figure 3). Simultaneously, the biogenic synthesis could face challenges in producing monodisperse NPs with fine tune particle size distribution [90]. Additionally, some nanoparticles produced by microorganisms can be less stable as opposed to those synthesized by chemical processes [91]. However, with the proper selection of the best biological candidates based on their inherited properties of growth rate and biochemical activities as well as parameters such as pH, temperature, process time, and reagent concentration, the biogenic processes can be improved with an attempt to obtain high quality and yield of biosynthesized NPs at the expanse of lower cost because of lack of organic solvents, thermal stabilizers, and expensive production techniques.

### 3.1. Biosynthesized NPs by Using Plants and Their Extracts

At a large scale, the synthesis methods utilizing plant extracts are comparatively easy, simple, and cost-effective as opposed to fungi or bacteria-mediated production and they present feasible methods and alternatives to conventional NP production methods. Because of the broad availability of biologically active plant extracts and their biodegradability, the biosynthesized NPs from plant extracts are receiving great interest. Plant materials like leaves, flowers, fruits, roots, seeds, etc. are available at all times and seasons at large volumes for large-scale synthesis while using them will not affect crop productivity.

Plants are known to possess the potential to accumulate some quantity of heavy metals at various parts, which makes them suitable for in vivo synthesis of metal NPs by absorbing soluble salts. The steps involved in NPs production include bio-reduction and nucleation of reduced ions, growth, and termination including the formation of the final shape of the nanomaterial [5]. Although the mechanisms of plant-mediated synthesis of NPs are still under continual research, it is known that organic matter plays a vital role in crystal growth and forming certain particle sizes [92]. Negatively charged groups concentrate positive ions from the solution, thus contributing to ion saturation to initiate the nucleation. The main functional groups involved in the reduction of metal ions are hydroxyl, amino, carbonyl, and methoxide, which electrostatically interact with the ions and lead to their reduction [93]. The ionic forms of metal can be easily detached from anionic parts because of the reduction process that renders them stable in the presence of plant extracts [94,95].

The major part of the research focused on the ex vivo production of NPs from plant extracts. The extracellular synthesis is advantageous because it excludes the presence of intracellular proteins and additional treatments with chemicals or ultrasonic cleaning. The synthesis parameters such as pH, temperature, salt concentration, etc. control the yield, size, rate of formation, and NPs’ stability [96]. For instance, the many metal ions absorbed on the surface of preformed nuclei will lead to secondary reduction and enlargement of NPs [97]. As the reaction temperature increases, the reduction rate rises and many metal ions are consumed for NPs formation, thus blocking the secondary reduction process on the surface of the preformed nuclei, which leads to small and highly dispersed NPs formations with increased yield [98]. Simultaneously, with the increase in reaction time the size of the metal NPs also increases due to aggregation. Under different pH conditions, desired size and shape uniformity can be obtained [99]. The growth rate of NPs increases with the increase of the reducing agents, but too many reducing agents may trigger a bridging effect among the produced NPs and their aggregation [100]. Additionally, the composition of the plant extract that contains a different concentration of phytochemicals is also an important factor in the bio-assembly. The phytochemicals responsible for the reduction and cupping of NPs are sugars, ketones, carboxylic acids, terpenoids, flavones, aldehydes, amides, alkaloids, etc. These constituents effectively activate the reduction mechanisms and reduce and stabilize the produced NPs. Some plants, such as cruciferous vegetable extracts, are also capable of the synthesis of bimetallic NPs in the form of core-shell structures of Au core and Ag shell [101].

Plants seem to be superior candidates for large-scale biomimetic synthesis of NPs because of the fast rate of formation and the variety in shape and size of NPs. Table 1 demonstrates some recent achievements of plant-mediated NP synthesis together with the particle characteristics and their application. It can be summarized that biosynthesis is simple, relatively rapid, and cost-effective. When used in the nanobiotechnology field, many plants containing pharmacologically active substances can support the action of treatment. However, when using plants, it is hard to maintain monodisperse in the NP population as well as reproducibility of the process [102]. Because of the presence of many organic compounds in the plant extracts, it may also be difficult to identify the exact reactive components. Another issue in photosynthesis is the use of plants of commercial value as reducing and stabilizing agents, which affects the efficacy of the synthetic procedure [103]. Challenges associated with unoptimized reaction process parameters and unexplored qualitative growth kinetics of NPs are also obstacles for the successful commercialization of phytogenic processes [103].

### 3.2. Biosynthesized NPs by Using Microorganisms

#### 3.2.1. Bacterial Synthesis

Bacteria are prokaryotic microorganisms. Metal-reducing procaryotic bacteria and actinomyces have been broadly used for the production of metal and metal oxide NPs because bacteria are easily genetically manipulated [127]. Bacteria are extremely adaptable, fast-growing, and have environmental abundance. Bacteria are also capable of surviving various stressful conditions, including the presence of higher concentrations of toxic metals [128]. When exposed to high concentrations of heavy metal ions, they use different defense mechanisms to overcome the stress conditions. Depending on the location of NP formation, the biogenesis of NPs can be either extracellular or intracellular by encapsulation in the cytoplasm. The mechanism behind the extracellular synthesis of NPs includes the aggregation of metal ions on the cell surface and the involvement of enzymes. The negatively charged polysaccharides containing phosphoric and carboxylic groups can bind to the positively charged metal/metal oxide ions via electrostatic interaction [129]. Moreover, because of the presence of negatively-charged amino acids, such as aspartic acid and glutamic acid, various enzymes and peptides are essential for the reduction of metal ions to NPs. The main enzymes are oxidoreductases, such as nitrate reductase, sulfate reductase, and cellular transporters [130,131,132]. For example, after the initial electrostatic interaction of the metallic ions with the cell wall of the bacteria, reduction and the bio-reduced metallic nuclei growth can be assisted by NADH (nicotinamide adenine dinucleotide) and NADH-dependent nitrate reductase that transferred electrons into NPs [130]. During the enzyme-assisted reduction, various amino-acid residuals can link to the metal or metal oxide ions and then reduce them to metal or metal oxide NPs [131]. Metal-reducing strains such as *Shewanella* have evolved a mechanism for electron transfer across the cell membrane via periplasm to the surface that involves c-type cytochromes from quinol at the inner membrane to metal oxide surface (Fe(III) oxide) at the outer membrane [132]. Other authors found that hemiacetal and aldehyde groups of exopolysaccharides in *E. coli* were involved as bio-reducing agents in Ag NPs production [133]. After contact with exopolysaccharides, metal ions are chelated and after that reduced and capped by the various groups of reducing sugars. Although actinomycetes were found to synthesize NPs via both intracellular and extracellular mechanisms, extracellular biomineralization was commonly used for commercial applications [13]. Among them, *Streptomyces* sp. is most widely utilized in pharmaceutical applications because 55% of the known antibiotics are produced by them.

Some magnetotactic bacteria have been used for the production of magnetosomes with magnetic radicals with controlled geometry and composition of the NPs [134]. The magnetosomes are organic-coated nanocrystals usually containing iron oxide NPs with an organic layer of bacterial phospholipid membrane that can be applied in cancer therapy, molecular imaging [135], or carrying drugs or other loads in cancer treatment [136].

The advantages of the bacterial-synthesized NPs are their production from impure materials and other feedstocks at ambient conditions and mild temperatures [137]. However, compared with plant synthesis, the bioproduction of NPs using microorganisms can be in some cases pathogenic to humans, and it requires precise control over cultivating and maintaining the microbial cells. Other main issues are the need for a mechanical breakdown of the cell wall, centrifugation, purification of NPs, and difficulty in controlling their geometry [138]. Compared with fungi, bacteria produce a much smaller volume of NPs because of their lower protein production [87]. The bacteria culturing is also tedious, the process conditions for bacteria growth are inflexible, and the reduction process is slow, lasting from hours to days [139].

#### 3.2.2. Fungal Synthesis

Fungi are easier to culture in laboratories and industries than microorganisms. Fungi are found to be efficient in the biogenesis of monodisperse NPs with well-defined morphology. Because of the existence of a large number of intracellular enzymes, they are thought to be more effective microorganisms in the production of metallic NPs compared to bacteria [140]. Fungi can secrete a large amount of red-ox active extracellular proteins, thus increasing the synthesis of NPs forming insoluble metal-protein conjugates. They were also found to have higher productivity and higher tolerance to metals because of the higher cell wall binding capacity of these ions with biomass and higher bioaccumulation ability [141]. Additionally, fungi possess reducing components, proteins, and enzymes (such as reductase) on their cell surface [142]. The polysaccharide chitin, a key ingredient in the fungal cell wall, was also found to be involved in heavy metal complexation and biogenic NP production [143].

Fungi can produce NPs intra- and extra-cellularly. For example, *Fusarium oxysporum* was found to produce Ag NPs by extracellular mechanisms with the action of NADPH nitrate reductase and anthraquinones [144]. The reduction of the metal ions to NPs is accompanied by the oxidation of NADPH to NADP^+^, whereas naphthoquinones and anthraquinones facilitate the reduction by a quinone-based shuttle [145]. Alternatively, NPs or metal ions may diffuse through the cell membrane and be reduced by red-ox systems in the cytoplasm. The change in pH in the medium can trigger the formation of NPs with different sizes since the changes in pH influence the acidic or basic nature of amino acids that participate in biogenic synthesis [146]. The incubation temperature, salt concentration, and incubation time also affected the size and rate of the extracellular bio-fabrication of Au NPs [147].

Extracellular synthesis is cheaper and simpler, while intracellular production provides NPs that should be additionally purified. Many fungi form mycelia, which provide higher surface area than bacteria for supporting fungal biomass-ions interactions. Filamentous fungi are preferred to bacteria because they are highly tolerant to metals, capable of extracellular NP formation, and easy to handle [148]. Fungal micelles can withstand pressure, agitation, flow, or other conditions compared to plant materials or bacteria. However, similarly to bacteria, the major drawback of fungi-produced NPs relates to biosafety and low rate for synthesis in comparison with plant extract. Some species, such as *F. oxysporum*, are pathogenic, which makes them a health concern, while others, such as Trichoderma fungus, are vastly utilized in food and medical application [149]. Attention should also be paid to the production of mycelia-free filtrates.

#### 3.2.3. Yeast Synthesis

These single-cell eukaryotes have been reported to be used in the successful biosynthesis of various metallic NPs because they possess various detoxication mechanisms. Yeasts are classified in the kingdom of fungi and, similarly to them, have an envelope composed of chitin, glycoproteins, and β-glucans that may participate in extracellular NP biogenesis. Intracellular reduction of metal ions occurs after the passive diffusion of metal salts followed by reduction mediated by the transport of reductive agents into the cell [150]. More focus is put on extracellular synthesis, since additional operations like ultrasound processing and chemical treatment are eliminated.

Silver-tolerant species such as *Saccharomyces cerevisiae* were usually used for the synthesis of Au [151] and Ag [152] NPs, but, recently, some other species have been adopted as biogenic NP producers. For example, Zhang et al. demonstrated a biosynthesis of Au NPs with hexagonal, spherical, and triangular morphology through the yeast *Magnusiomyces ingens* LH-F1 [153]. The genetically engineered yeast strain *Pichia pastoris* has also been used for the biogenic fabrication of Ag NPs [154]. The yeast strain was transformed with an upregulated metal-resistant gene from *Mucor racemosus* produced cytochrome b5 reductase enzyme. Thus, the yeasts were capable to produce stable Ag NPs with a size of 70–180 nm. Not only commonly produced Ag NPs but also Pd NPs with hexagonal form and a size of 32 nm were synthesized by *Saccharomyces cerevisiae* [155]. The yeast *Saccharomyces cerevisiae* and its three mutants with deleted genes were also used for the production of ZnO NPs with an average size of 20 nm [156]. Likewise, anatase TiO_2_ NPs with an average size of less than 12 nm were biosynthesized through incubation with Baker’s yeast [157]. They showed a significant antibacterial effect on selected pathogens especially against Gram-positive bacteria in the presence or absence of sunlight exposure. Easy control of mass production and rapid growth of yeasts make them preferred microorganisms for NP biosynthesis.

**Figure 4:**
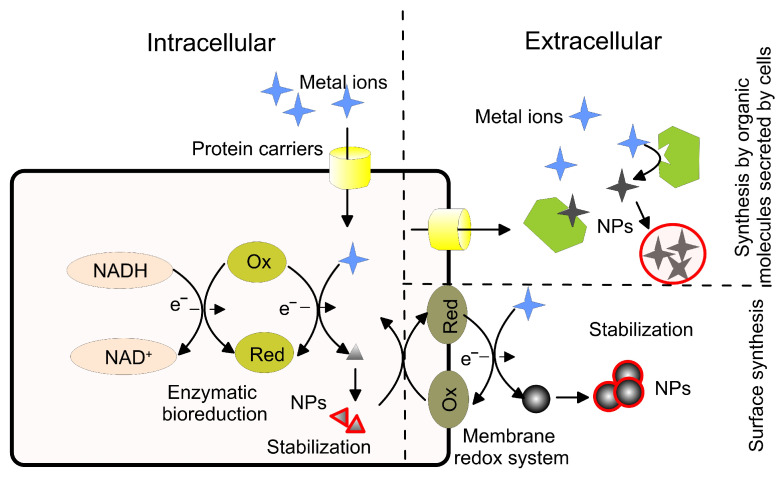
Intracellular and extracellular mechanisms of NPs biosynthesis. The intracellular synthesis can take place by cofactors, enzymes or other proteins. The extracellular process can be mediated by functional groups of macromolecules on the cell membrane/cell wall or by secreted biomolecules.

A schematic presentation of the synthesis process by biogenic routes that use microorganisms is shown in Figure 4.

The biogenetic synthesis from microbes including bacteria, yeast, and fungi may suffer from complications related to extraction, purification, and cell separation from the surface of NPs, which may cause serious adverse effects on living cells in the biomedical application [158]. Extra steps such as detergent treatment or ultrasonication, which further increase the price of production, are mandatory for the separation of NPs in the intracellular biogenesis route [159]. Additional limitations can be operational impediments such as (1) longer time (up to 120 h) for the reduction reactions; (2) hard retrieval of NPs from biomass; (3) selection of the best organism for the biosynthesis and biocatalyst state; and (4) picking the optimal conditions for cell growth and enzyme activity [103]. Additionally, problems related to the commercialization of the biosynthesis protocol at an industrial level are the main expected obstacles [103].

### 3.3. Virus Synthesis

Viruses are unicellular organisms that use the replication machinery of the host. Their structure consists of nucleic acid (DNA or RNA) surrounded by a protein shell, and/or lipid envelope. Viruses present in host cells can also be used to generate biogenicNPs. They are considered valuable biogenic resources for the synthesis of NPs in organized assemblies including several morphologies. The outer capsid proteins of the viruses offer a very reactive surface for interaction with metallic ions [13]. Hydroxyl groups in tyrosine and carboxyl groups in asparagine and glutamine or indole groups in tryptophan may participate in the reduction of the metallic ions. The sequence configuration has the potential to attract metal ions and controls the size and morphology of NPs [160]. Pathogens such as animal and plant viruses and bacteriophages have been used for nanobiotechnological purposes because of their structural and chemical stability, lack of toxicity and pathogenicity in humans, and ease of production [158]. Plant viruses are easy to cultivate and non-pathogenic to humus and, therefore, can be used to produce NPs with the nanomedical application. For example, a high yield of Au and Ag NPs with small sizes was reported to be biosynthesized when a low concentration of *Tobacco mosaic virus* (TMV) was added to *Hordeum vulgare* (barley) or *Nicotiana benthamiana* plant extract as opposed to those without viral particles [161]. Recently, Thangavelu et al. produced Au-Ag composite NPs through plant pathogenic squash leaf curl China virus [162]. The hybrid NPs biosynthesized through virus nanobiotemplate (32 nm) showed good biocompatibility. Bacteriophage-inspired biogenesis of Au NPs through a rare bacteriophage (*Podoviridae* family, C3 morphotype), also used as a reducing agent, revealed the presence of NPs in the form of spheres, hexagons, triangles, rhomboids, and rectangles, with a size ranging from 20 to 100 nm [163]. Viruses can be used for the production of nanocomposites with metal NPs useful for target drug delivery and cancer therapy [164]. For example, retargeted adenoviral vectors covalently bound to AuNPs have been also used as selective agents for the delivery of NPs to tumor cells [165]. However, the involvement of host organisms for protein synthesis and a small amount of research on large-scale applications still limit NPs’ production by viruses. Moreover, a few viruses displaying nucleation properties have been recently used as bio-templates for NPs synthesis [162]. Selecting the optimal process conditions for virus template synthesis and identifying the types of amino acids involved in the surface mineralization process are also crucial for the optimization of nucleation and growth of NPs [162].

### 3.4. Algae Synthesis

Algae are aquatic eukaryotic photosynthetic organisms that lack the multicellular structure of the plant. They can vary in size from microalgae to giant macroalgae. Microalgae are known to transform metal ions into NPs in a biogenic way, including active compounds in the cell wall such as laminarin that contain reactive groups [166]. Cyanobacteria strains grow faster than plants and are suitable for the eco-friendly and time-saving synthesis of NPs at ambient temperature. The interaction between Ag-containing solution and biomass allows the formation of Ag NPs without degradation of the biomass [167]. Microalgae-like diatoms (*D. gallica* and *N. atomus*) were also capable of producing Au NPs and Au-silica NPs [168]. It was also discovered that the formulation of Ag NPs includes extracellular compounds (polysaccharides) participating in the cell-free culture media [169]. Al-Katib et al. [170] found that the presence of specific functional groups in proteins is important in cupping and stabilizing during the extracellular synthesis of Ag NPs in *Gloeocapsa* sp. In addition to microalgae, biosynthesis of ZnO NPs by marine brown seaweeds (*Turbinaria conoidea* and *Padina tetrastomatica*) [171] was also reported. *Tetraselmus kochinensis* has also been shown to produce intracellular Au NPs [172]. The marine algae are found to be suitable for many biomedical applications, such as anticancer, antiviral, antioxidant, etc. agents, because the biosynthesized NPs contain different bioactive compounds and secondary metabolites [149]. Algae are characterized by easy culturing, high growth rate and biomass productivity compared to other microorganisms, which makes the biogenic algae-mediated process cost-effective. However, similar to microorganisms, downstream processing for the extraction of intracellularly produced NPs is still challenging while extracellular biogenesis is greatly influenced by cultivation conditions [173]. Various challenges, such as strain selection, low reaction rate and yield, size control and monodispersity, and cytotoxicity to non-target cells, still limit the scaling up of the bio-process to a commercial scale [174]. Limited information is also available on NPs biogenesis using microalgae, which hinders their applicability [175].

### 3.5. Biosynthesized NPs by Using Enzymes and Biomolecules

The synthesis of NPs using fungi or microbial systems is a slow process with a low yield that may result in the production of polydisperse NPs. For that reason, different enzymes and their metabolites such as polysaccharides, peptide chains, carbohydrates, nucleic acids, etc., are utilized as reducing and capping agents for the production of metal/metal oxide ions into NPs. Due to the excellent biocompatibility and a great number of functional groups, biopolymers (both native and modified) are vastly explored for the biogenesis of metallic NPs. There are three main approaches for the synthesis of biopolymer-supported NPs: (1) impregnation of the polymer with ion salt followed by its in situ reduction by hydroxyl groups of biopolymers; (2) impregnation with ion salt and addition of external reducing agent; and (3) synthesis of colloidal NPs and adoption into biopolymer branches [176]. The first one is the most convenient and frequently used. Several natural compounds such as cellulose [177], chitosan [178], and silk fibers [179] have been reported as reducing agents of Au^3+^ utilized for the biosynthesis and stabilization of Au NPs. The biogenesis of TiO_2_ NPs using starch [180], cellulose fibers [181], albumen [182], and lysozymes [183] has been reported. Although different polysaccharides, such as cellulose, chitosan, dextran, and starch, have been used for the production of metallic NPs, they require additional stabilizing. In contrast, pullulan was found to be effective in the reduction and stabilization of Au NPs produced from HAuCl_4_ solution [184]. An interesting study by Safat et al. demonstrated cerium oxide NP synthesis obtained from marine oyster extract used as a bio-reducing and capping/stabilizing agent. The marine biosynthesized NPs with a size of 15 ± 1 nm had no cytotoxic effect on normal cells [185]. Therefore, a great variety of polymers with several functional groups can produce NPs. However, all these polymers should be precisely examined for their reducing activity, efficacy, and bio-toxicity. The difference in concentration of active molecules because of external factor changes can affect the biosynthesis procedure [186].

With the help of commercially available enzymes in pure form, the production and purification of biogenic NPs can be eased. For example, Maddinedi et al. proposed the use of a natural enzyme, diastase, to synthesize Au NPs with a spherical shape and size of 9.7 nm [187]. With the increasing volume of enzymes, smaller NPs were produced. Similarly, an enzyme, Ia bacteriocin, belonging to nisin peptides, was also used for the biosynthesis of Au NP with a small size (25 nm) [188]. The enzyme consisting of 34 amino acids was obtained from *Lactococcus lactis* susbp. Arib et al. also reported a one-step enzyme-based synthesis of hybrid Au NPs using manganese superoxide dismutase protein in the presence of a zwitterionic sulphonic acid-based buffer as a reducing agent [189]. It follows that the enzymatic approach of NP synthesis proposes superior characteristics and easy purification of the obtained NPs but it is more expensive and time-consuming.

Table 2 sums up the biosynthesized NPs of various sources together with their characteristics and application.

## 4. Therapy and Drug Delivery of Biosynthesized NPs

Green chemistry focuses on the development of biogenic NPs that provide a reduction of harmful and toxic moieties of physiochemical processes and enables lower dose prescription of drugs during treatment. On the one hand, biogenic NPs themselves showed remarkable properties as antimicrobial and anticancer compounds [190,202,211,214]. However, when loaded with drugs, their efficiency can be additionally enhanced. On the other hand, to achieve target delivery and seize the rapid degradation of drugs, the concept of controlled drug-delivery systems (DDS) that overcome both physicochemical and biological barriers has been developed [223]. Because of the large surface area, NPs have drawn significant attention as potential DDS that can bind, adsorb, or entrap different drug molecules. The drug intended for release is usually directly bound to the nanocarrier and the time for release is of prime importance because it should not dissociate before reaching the target. The small NPs can pass through the extracellular matrix and assess layers that normal drugs are unable to reach [223]. Because of their unique chemical and physical properties that completely differ from their bulk counterparts, using NPs as potent DDS can improve the loading capacity and resides in sustained drug release, higher bioavailability, and enhanced target intracellular penetration [224]. It was also found that nanocarriers containing natural compounds may be useful for delaying the development of drug resistance and stimulating the low response to treatment with conventional medicine [225].

The targeting can be active (when peptides or antibodies are coupled with drug-delivery vectors to link to the receptor structure) or passive (when circulating in the bloodstream the nanosystem is passively attracted by changing factors such as temperature, pH, molecular folding, etc.). Therefore, the drug release can follow the stimuli-controlled release, chemical or physical reactions, etc. [226]. The selection of a particular metallic nano-vector is based on the physicochemical features of the drugs. Moreover, natural materials can provide different solutions for improving drug loading challenges. By presenting different active groups, chemicals such as covalent or hydrogen bonds, or physical interactions, such as electrostatic, may take place between drug polarities and capping or nanomaterials. Some drugs themselves, such as vancomycin and doxorubicin, also come from natural substances.

### 4.1. Antitumor Activity

Recently, traditional cancer drugs not only inhibit the growth and kill tumor cells but also demonstrate obvious cytotoxicity to healthy cells and trigger tolerance of the body during long-term use. Developing tolerance to commonly used therapeutic drugs, such as docetaxel, paclitaxel, epiamicyn, adriamycin, etc., requires increasing the dosage that induces more pronounced adverse effects [227]. Therefore, the necessity of new drug development is an obvious and urgent task. Because of their large surface area, high drug-loading capacity, and tumor targeting, nano-drugs have attracted much attention with superiority in tumor treatment. Many studies, some of which are reviewed below, have shown that biogenic NPs themselves indicate significant chemotherapeutic effects on various tumor cells. The main mechanisms accounting for antitumor activity of biogenic NPs are (1) apoptotic pathways depending on increased levels of ROS that led to oxidative stress and DNA fragmentation; (2) interference with macromolecules such as proteins and DNA resulting in disturbance of cell functions; and (3) interaction to cell membranes thus changing its permeability and causing mitochondrial dysfunction [100]. The scientists mainly focus on biogenic NPs synthesized by plants because phytoconstituents could exert activity against cancer cells through the production of ROS participating in phagocytosis, regulation in cell proliferation, and intercellular signaling [228]. Furthermore, phenols and polyphenolic compounds were discovered to be associated with atherosclerosis and inhibition of cancer [229].

#### 4.1.1. Biosynthesized Metal NPs in Cancer Therapy and Delivery of Antitumor Drugs

Some nanomaterials have inherited antitumor activity and can be directly used as drugs. They can work as cytotoxic agents due to their physical-chemical and surface properties. NPs act as nanocarriers to passively target the tumor via enhanced permeability and retention effect (EPR) or actively target solid tumors by ligand-receptor interactions [230]. Active targeting includes the conjugation of different molecules, such as DNA, peptides, and antibodies, to target specific cells [4]. At present, Au NPs are known to be efficient as pharmaceutical drug carriers that improve antitumor efficiency. Au NPs themselves, with a size of 15–35 nm, extracellularly developed by using *Paracoccus haeundaensis* (marine bacterium), have demonstrated non-toxicity of human cells while preventing the growth of AGS and A549 cancerous cells at different concentrations [231]. Au NPs synthesized from *Solanum xanthocarpum* leaf extract also demonstrated induced apoptosis of the nasopharyngeal cancer C666-1 cell line with a decline in both cell viability and colony formation upon treatment [232]. The cell death was proved to be caused by autophagy and mitochondrial-dependent apoptotic pathway. Similarly, the amalgamation of Au NPs with *Scutellaria barbata* plant extract possessed effective anticancer activity against the pancreatic cell line PANC-1 [233]. The authors found upregulation of the expression levels of Caspase-3, Caspase-9, and Bax genes while the gene expression of Bid and BCl-2 genes was downregulated. Likewise, Zhang Y et al. reported that verboascoside (a major bioactive constituent of the Tsoong herb)-loaded Au NPs exhibited apoptosis in both in vitro (K562 tumor cells) and in vivo studies with mouse models [234]. The intravenous injection of the NPs effectively inhibited the growth and induced apoptosis of tumor cells. Additionally, the well-adsorbed tumor cells Au NPs were susceptible to radiation in the near-infrared region which is frequently used in heat therapy for selective cancer elimination [235].

Specific targeting of cytotoxic drugs to malignant cells appeared to be a promising strategy for reducing the adverse side effects and efficacy of anticancer treatment. Nanocarrier delivery systems can propose some advantages in tumor treatment for better efficacy, such as increased solubility of the drug, reduced interaction with non-target tissue, and avoiding drug degradation thus reducing adverse reactions [236]. By using trisodium citrates capping and reducing agent, Au NPs with an average size of about 22 nm were biosynthesized and functionalized with PEG and folic acid and loaded with the anticancer drug Docetaxel [237]. As compared with Docetaxel, the functionalized Au NPs demonstrated good potential for target drug delivery when tested by using the A549 cell line (lung cancer cell line). By using chitosan as a reducing and capping agent, Malati et al. demonstrated that Au NPs conjugated with rifampicin ensured the biocompatible controlled release of the drug in the body [238]. Modified by activated folic acid ligands, biosynthesized Au NPs loaded with chlorambucil showed higher toxicity towards HeLa, RKO, and A549 cell lines as compared to Au NPs and chlorambucil alone [239]. The release rate of the drug was faster at lower pH (5.4) than at pH 6.7 and pH 7.2. Therefore, the synthesized biogenic NPs demonstrated potential for pH-sensitive drug delivery in a tumor microenvironment where the pH value varied between 4.5 and 6.5 and slowed drug release in the bloodstream (pH 7.2).

Alqahtani et al. reported the biosynthesis of Ag NPs with a size of 1 to 40 nm using lichens, namely, *Xanthoria parietina* and *Flavopunctelia flaventior*. They demonstrated the antitumor activity of the biogenic Ag NPs against human colorectal (HCT 116), breast (MDA-MB-231), and pharynx (FaDu) cancer cells using a routine MTT assay. The biogenic Ag NPs exhibited higher cytotoxicity towards human colorectal and pharynx cancer cells than breast cancer cells [240]. Doxorubicin is a commonly used chemotherapeutic agent and several attempts have been made to tether them with metal nanoparticles. These efforts are continuing since most cancer cells have developed resistance to doxorubicin. By using curry leaves and neem extracts, Thirumurugan et al. [241] formulated Pt NPs and Ag NPs with encapsulated Doxorubicin (Dox). In vitro tests with MCF-7 cell lines revealed that a better inhibition value (IC_50_) against MCF-7 demonstrated Dox-coated Ag NPs than Dox-coated Pt NPs. Simultaneously, the toxicity of both drug-loaded nanomaterials was found to be concentration-dependent. It follows that the increased pharmacokinetic characteristics of the engineered nanocomposite result in better therapeutic effects. Phytogenic monodisperse (below 10 nm) Pt NPs and Pd NPs synthesized by using the medical plant *Gloriosa superba* tuber extract showed high anticancer activity against the human breast adenocarcinoma MCF-7 cell line [242]. Pt NPs showed higher (49.6%) anticancer activity than Pd NPs (36.3%). It was confirmed that the predominant mechanism in anticancer activity was apoptosis-induced due to the externalization of phosphatidyl serine and membrane blebbing. However, biosynthesized Pd NPs containing phenols and flavonoids acquired from white tea (*Camellia sinensis*) extract with a size of 6–18 nm were found to be more antiproliferative towards human leukemia (MOLT-4) cells than pure white tee extract, doxorubicin or cisplatin [243]. The anticancer activity of the biosynthesized NPs was mediated through the induction of apoptosis and G2/M cell cycle arrest. Recently, Prakashkumar et al. reported the preparation of neem gum-coated Pd NPs that exhibited anticancer and antimicrobial activities. The authors biosynthesized Pd NPs using an aqueous leaf extract of *Orthosiphon stamineus*. The neem gum-coated Pd NPs also exhibited an anticancer effect in A549 cells via ROS-induced MMP loss activating apoptosis. The authors also demonstrated the biocompatibility of neem gum-coated Pd NPs as they did not exhibit any cytotoxicity or hemolysis, thus making them an ideal candidate for antimicrobial and cancer therapy [244].

#### 4.1.2. Biogenic Metal Oxide NPs in Cancer Therapy and Delivery of Antitumor Drugs

ZnO is considered to be one of the five metal oxides that are recognized as safe and approved by FDA material. Biosynthesized ZnO NPs using *Albizia lebbeck* stem bark extracts and an average size of 66.3 nm and spherical morphology revealed a strong cytotoxic effect on MDA-MB 231 and MCF-7 breast cancer cell lines in a concentration-dependent manner [122]. Similarly, biogenic porous rod ZnO NPs synthesized by using *Borassus flabellifer* fruit extract and an average size of 55 nm were utilized for drug delivery of Doxorubicin [245]. The conjugate had low systemic toxicity in a murine model and high loading capacity and therapy efficacy. Drug-loaded ZnO NPs indicated a pH-responsive release of drug preferentially to malignant HT-29 and MCF-7 cells. Not only plant-synthesized but also fungus-mediated produced ZnO NPs also demonstrated anticancer activity. Through *Aspergillus niger*-mediated synthesis, ZnO NPs with a size of 39–115 nm showed significant cytotoxic effects against rapidly dividing HEP-2 cell lines [246].

Fadeel et al. reported a novel biosynthesis of TiO_2_ NPs that was prepared from *Aloe vera* leaf extracts and was utilized as a drug delivery agent for doxorubicin. The authors demonstrated through in vivo studies that the presence of biogenic TiO_2_ NPs enhanced the anticancer activity of doxorubicin in Ehrlich tumor-bearing mice in comparison to anticancer activities of doxorubicin-loaded liposomes and pure doxorubicin aqueous solutions [247]. Nageshwara et al. reported biogenesis of Ag-doped TiO_2_ spherical nanoparticles with *Acacia nilotica* extraction. The authors demonstrated that Ag-doped TiO_2_ NPs induced cytotoxicity in human breast adenocarcinoma cell lines, thereby proving to be anticancer agents with their utilization as drug delivery agents [248].

Biosynthesized spherical copper oxide NPs (577 nm in size) produced by using *Ficus religiosa* leaf extract showed stable anti-cancer effects against A549 tumor cells [249]. The authors attributed the apoptotic effect of copper oxide NPs to the generation of ROS triggering disruption of mitochondrial membrane potential.

Because superparamagnetic iron NPs (SPIONs) loaded with a drug can be guided with the help of an external magnetic field to the desired site, they were found to be a promising agent in cancer therapy and magnetic resonance imaging (MRI). To reduce their toxicity and agglomeration, these NPs are generally coated with non-toxic and biologically active compounds [250]. For that purpose, Tyagi et al. [251] have biosynthesized SPIONs via biogenic techniques and further coated them with tamoxifen-conjugated bovine albumin. The NPs were efficiently internalized into breast cancer cell lines (MCF-7 and T47D) and effectively reduced cell proliferation with IC50 values of 5 ± 0.4 μM and 6.3 ± 0.2 μM in MCF-7 and T47D, respectively. The SPIONs were confirmed to be safe for use as DDS by acute toxicity studies in the rat. Likewise, cellulose nanofiber composites with a size of 62.5 nm with Fe_3_O_4_-Ag_2_O NPs loaded with etoposide and methotrexate demonstrated a slow and steady release of methotrexate (63.8%) and etoposide (78.9%) together with non-toxicity which made it suitable for anticancer application [252].

**Figure 5:**
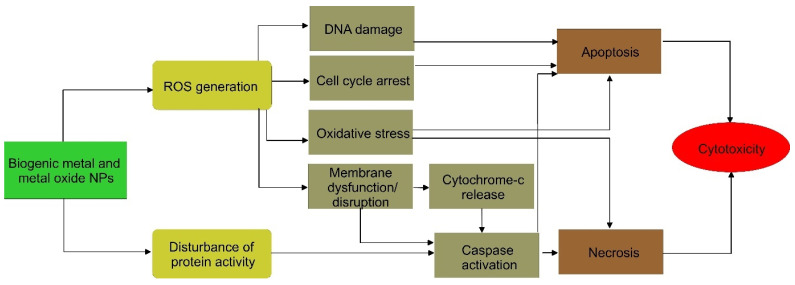
Cytotoxic mechanisms of action of biogenic NPs that trigger apoptosis or necrosis in cancer cells.

Table 3 shows some examples of biosynthesized metallic and metal oxide NPs, their characteristic features, tested objects, and the main outcomes of their antitumor activities. The cytotoxicity triggered by biosynthesized NPs is based on their type, shape, size, and surface chemistry. The mechanisms of action of biogenic NPs are still not fully studied but based on the results reviewed in this study, it can be concluded that they include both ROS generation and interaction with macromolecules. The formation of ROS by the NPs is an important mechanism in toxicity that interferes with the equilibrium between oxidant and antioxidant species thus causing changes in intercellular activities. The latter mainly includes DNA damage, cell cycle arrest, induction of oxidative stress, and membrane disruption (Figure 5). Both mechanisms activate apoptotic pathways or trigger necrosis. Most of the biogenic NPs achieve both passing targeting and obvious antitumor effects while drug loading for cancer therapy can additionally improve the efficacy and reduce the death rate.

### 4.2. Biogenic NPs for Antimicrobial/Antifungal Therapy

The development of resistant bacteria and fungi against antibiotics is a globally increasing problem whose solution lies in finding novel materials to alleviate resistant strains. Metals such as Ag, Cu, Au, Fe, and their nano-metallic forms exhibit numerous biocidal activities against resistant Gram-positive and Gram-negative bacteria and eukaryotes. Biosynthesized NPs are known to present higher antimicrobial activity as opposed to commercial or chemically produced NPs because of the medical properties of some plants [276]. Therefore, the activity of biogenic metal NPs depends on the type, size, shape, charge, and capping, as well as genus and species [277]. For example, Hamed et al. demonstrated antimicrobial and antibiofilm activities of Ag NPs synthesized from actinomycetes extracted from the marine sponge *Crella cyathophora*. These biogenic Ag NPs showed antimicrobial activity towards various Gram-positive and negative bacterial strains particularly *P. aeruginosa* and *E. cloacae*. Further, Ag NPs derived from marine actinomycetes exhibited significant biofilm inhibition against *B. subtilis*, *S. aureus*, and *P. aeruginosa* [278]. Shabaan et al. reported the biogenesis of silver, selenium, and zinc oxide NPs from *Streptomyces enissocaesilis*, which also exhibited antimicrobial activities against the various standard and resistant bacterial isolates [279]. Small-sized (2–7 nm) Pt NPs biosynthesized from a plant extract of *Taraxacum leavigatum* showed strong antibacterial activity against *P. aeruginosa* and *B. subtilis*, which have strong defensive mechanisms against various antibiotics [280]. Phytosynthesized NiO NPs reduced with garlic and ginger increased the bactericidal activity against multi-drug resistant Staphylococcus aureus [281]. ZnO NPs with an average size of 66.25 nm also biosynthesized with *Albizia lebbeck* stem bark extracts and revealed strong antibacterial potential against *Bacillus cereus*, *S. aureus*, *E. coli*, *Klebsiella pneumonia*, *Salmonella typhi* pathogens [122]. Likewise, by using bark extract of *Acacia ceasia* (L.) Wild, ZnO NPs with an average size of 32.32 nm and hexagonal morphology have been synthesized by Ashwini et al. [282]. The biomimetically synthesized ZnO NPs exhibited strong antibacterial activity against both gram-positive (*S. aureus*) and, especially, against gram-negative (*E. coli*) bacteria strains at all tested (250, 500, and 1000 μg) concentrations.

Several biosynthesized NPs have shown noteworthy growth inhibitory results against both bacteria and fungi. For example, biosynthesized iron NPs from *Acacia nilotica* seedless pods with an average size of 230 nm inhibited the growth of *E. coli*, *S. aureus*, *Salmonella*, *Marsa*, and *Candida* [283]. High inhibitory activity, especially against *E. coli* bacteria and *T. harzianum* fungal strain, was demonstrated by biosynthesized spherical CuO NPs (5–13 nm in size) by using *Syzygium alternifolium* stem bark [284]. However, the zone of inhibition was lesser in fungi as opposed to bacteria due to the difference in the structure of the cell walls of both organisms.

The cell membranes of bacteria are made up of peptidoglycans, which are less firm, and the passage of NPs is comparatively easy, especially in the case of gram-negative bacteria. In prokaryotes, multiple mechanisms are efficient in the antimicrobial activity of biogenic NPs. The biogenic NPs exert their antimicrobial activity due to releasing metal ions, interaction, and damage of the cell membrane, formation of pits, and fragmentation of the cell membrane [285]. Thus, the membrane permeability and intercellular communication are interrupted. NPs can interact with thiol groups and phosphorus of proteins and DNA thus disturbing the metabolic processes like DNA replication, protein synthesis, respiratory chains, etc., and causing cell death [286]. Some NPs can trigger the production of ROS thus causing phospholipid oxidation and the collapse of internal RNA, DNA, and proteins [287]. In thick cell wall microorganisms, such as gram-positive bacteria, moderate antibacterial activity is usually observed, since the cell dislocates the NPs by a transport system.

The fungal cell walls are made of chitin having N-acetylglucosamine and a nitrogen group which is firmer to allow the passage of NPs. Antifungal activity of metallic NPs is triggered by interaction with the cell wall and membrane diffusion of metallic ions which can inhibit β-glucan synthase or N-acetylglucosamine which are important components of the cell wall [288]. The induction of ROS and the initiation of oxidative stress cause interaction with different macromolecules such as DNA, RNA, and proteins that can lead to cell death [289].

**Figure 6:**
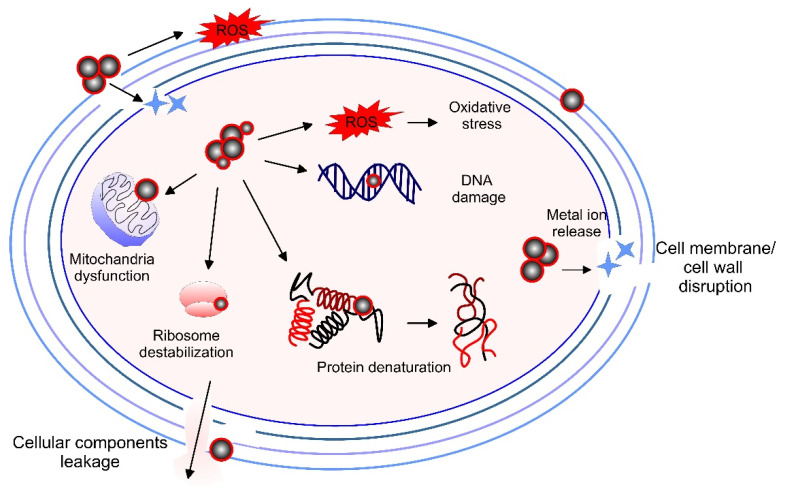
Mechanisms of antimicrobial activity of biosynthesized NPs.

A schematic representation of the mechanisms of antimicrobial activity of biosynthesized NPs is presented in Figure 6. They include direct contact of the NPs with the cell wall that damages the surface structures and penetration inside the cell. Additionally, the biogenic NPs can indirectly interact with the inside and outside cell environment generating ROS and metal ions that further damage the cell wall, change the permeability of the membrane, and affects protein, ribosome, and DNA functions. The biosynthesized NPs can also directly interfere with macromolecules, ribosomes, and mitochondria, thus ultimately triggering cell death by both mechanisms.

When biogenic NPs were loaded with biocidal drugs, synergetic effects or enrichment of biocidal properties were observed. For instance, Ag NPs with an average size of 9.8 nm that were bio-assembled by cell-free supernatant of *Delfitia* sp. strain showed increased efficiency against various pathogenic candida strains when conjugated with antifungal drug Miconazole [290]. The authors explained the fungicidal activity of the nanocomposite with inhibition of ergosterol synthesis and biofilm formation by increasing ROS levels. Furthermore, bio-synthesized by *Chlorella vulgaris* composites of biogenic CuFe_2_O_4_@Ag NPs loaded with Ciprofloxacin demonstrated high antibacterial activity against multi-resistant Staphylococcus aureus [291]. The synergetic effect of biosynthesized Ag NPs with Azithromycin and Clarithromycin against microorganisms causing dental caries and periodontal disease (such as *Lactobacillus acidophilus*, *Staphylococcus aureus*, *Micrococcus luteus*, *Streptococcus mutans*, *Bacillus subtilis*, *E. coli*, *Pseudomonas aeruginosa*, *Candida albicans*) was also reported by Emmanuel et al. [292]. All of these findings might be useful for determining if the potential efficacy of biosynthesized NPs of various types, sizes, and shapes might be boosted if different species are used for biogenesis. The effect of biogenic NPs on many bacteria is even better than that of bactericides or antibiotics. Most of the outcomes underline that these alternative antibacterial biogenic agents can prevent the growth and spread of microorganisms and alleviate microbial disease illnesses.

### 4.3. Anti-Inflammatory Activity

Inflammation is a response mechanism of the body to various external harmful factors. Mechanisms such as a gathering of macrophages and killer cells and the synthesis of cytokines, such as IL-1, IL-1β, IL-6, TNF-α, etc. in the desired site, develop the onset of inflammation [293]. Mild inflammation has a positive effect on living tissue for preventing the spread of pathogens and limiting the lesions. On the contrary, severe inflammation processes can cause denaturation or necrosis of organs, and can lead to fluid accumulation and mutual adhesion between organs, thus harming their normal functionality. Therefore, anti-inflammatory drug application is important in some disease treatments. The usually used steroidal and non-steroidal anti-inflammatory drugs have adverse effects and cause harmful reactions, which limit their application. Additionally, during acute inflammation, the drug efficacy is delayed because of slow absorption [294].

**Figure 7:**
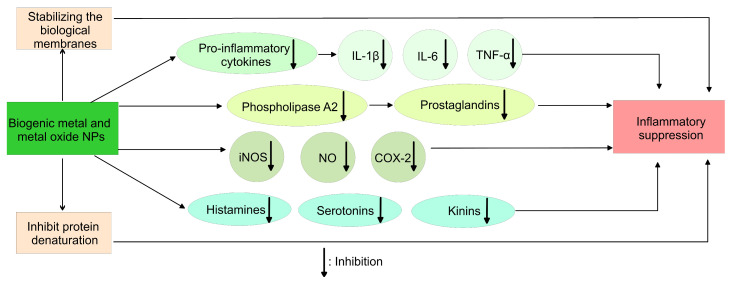
Anti-inflammation mechanisms of biosynthesized metal and metal oxide NPs.

Biosynthesized nano-based formulations are proven in developing antitumor drugs by blocking pro-inflammatory cytokines and ROS scavenging mechanisms and inhibiting different signal pathways. For example, silver NPs generated from *Selaginella myosurus* aqueous extract showed in vitro and in vivo anti-inflammatory potential by inhibiting protein denaturation. In the Carrageenen-induced rat hind paw edema model, the Ag NPs interfered with inflammatory mediators like histamines, serotonins, prostaglandins, kinins, etc., and blocked their action [295]. Likewise, gold NPs synthesized from *Prunus serrulate* fruit extract suppressed the production of pro-inflammatory cytokines and reduced the expression of inflammatory mediators such as nitric oxide (NO), prostaglandin E2, inducible nitric oxide synthase (iNOS) and cyclooxygenase-2 (COX-2) in RAW264.7 cells [296]. Phospholipase A2 enzyme is known to participate in the primary way of producing prostaglandins. Biogenic ZnO NPs produced from *Lantana camara* flower extract with an average size of 21.4–27.2 nm were found to inhibit phospholipase A2 activity via binding Zn^2+^ ions to its active site [297], thus performing their anti-inflammatory activity. Similarly, biosynthesized ZnO NPs employing leaf extract of *Pelargonium odoratissimum* (L.) with an average size of 34 nm showed a concentration-dependent anti-inflammatory effect via stabilizing the biological membranes, thus preventing the release of active mediators of inflammation and lytic enzymes [298]. A scheme summarizing the anti-inflammatory action of biogenic NPs is presented in Figure 7. Consequently, the development of biogenic nano candidates as an alternative clinical approach for anti-inflammatory treatment requires an understanding of the regulatory pathways and molecular mechanisms triggered by these nanomaterials.

### 4.4. Wound Healing Properties

Wounds are classified into acute and chronic based on healing time and other complications [299]. During the healing of various cells, growth factors, cytokines, coagulation, and vascularization growth factors intricate each other. Current therapies involve applying steroidal, nonsteroidal, and chemotherapeutic drugs with various side effects or therapies such as dressing materials, vascular surgery, hyperbaric oxygen therapy, etc. With the advance of the nano concept and knowledge of the significant antibacterial and anti-inflammatory properties of biogenic NPs, these nano-substances were found to be appropriate to facilitate skin wound healing. Predominate bacteria found during the early stages of infections are Gram-positive (like *S. aureus*, *S. pyogenes*), whereas Gram-negative (such as *E. coli*, *P. aeruginosa*) are found later when a chronic wound develops [300]. When the immune system is unable to remove the pathogens, the wound-healing process is disrupted [300].

When using biogenic NPs, wound therapy could be based on nanomaterials acting in the manner of drugs or biogenic nano-vehicles for delivery of agents to repair wounds. For example, *Lindera strychnifolia* extract produced Ag NPs with a 15.7 nm average size and showed the highest % (64%) of wound closure among seven biosynthesized Ag NPs determined by the scratch method on NH3T3 cells [301]. Magnetic (Fe_2_O_3_) NPs with 15–30 nm size biosynthesized by using Aloe vera extract in isolated bacterial nano-cellulose (BNC) were able to promote wound healing of human dermal fibroblast cells after 48 h [302]. The Fe_2_O_3_/BNC nanocomposite reduced the expression of microRNA (miR-29b and miR-29c) thus causing an increase in gene expression of other genes (TGF-β1, CTNNB1, MMP2, MMp9, WNT4), which resulted in faster wound healing. Similarly, *Barleria gibsoni* leaf extract mediated synthesis of ZnO NPs with sizes between 30 and 80 nm improved substantially the wound healing efficacy in male albino Wistar rats [303]. The wound contraction percentage of the nano-ZnO gel formulation reached above 98% on the 20th day as compared with the control group, thus avoiding wound chronicity. Sankar et al. confirmed that the *Ficus religiosa* leaf extract-tailored copper oxide NPs demonstrated both substantial inhibition activity against human pathogenies strains and enhanced wound healing activity in Wistar Albino rats [304].

To prevent would infections, various dressing materials with bacteria barrier properties, the ability to promote wound healing, and antibacterial potential have been designed [305]. In this regard, promising candidates are NP-loaded wound dressings that can reduce wound contraction time and wound infections without substantial side effects [306]. Such an approach includes the development of biodegradable and porous polymer membranes loaded with biogenic antimicrobial agents to promote healing and inhibit pathogens [307]. For example, Augustine et al. used photosynthesized Ag NPs through black pepper (*P. nigrum*) to incorporate them in electrospun polycaprolactone (PCL) membranes, and provided antimicrobial properties of the dressing composite material [308]. The membranes exhibited higher mechanical properties and antibacterial activities against both *E. coli* and *S. aureus* [309]. Moreover, the same research group utilized Ag NPs photosynthesized by *Mimosa pudica* to obtain electrospun biodegradable polyvinyl alcohol (PVA) membranes. The latter demonstrated good mechanical strength, wound fluid uptake, blood compatibility, cytocompatibility, and antibacterial potential. At optimum Ag NP composition, the composite membranes booted the wound healing in an in vitro wound contraction model.

Although the exact mechanism of wound healing is not yet well understood, its initial reaction is supported by inhibiting the proliferation of the microbial population. Therefore, the use of biogenic metal or metal oxide NPs with intrinsic antibacterial activity is a promising approach. Further, the biogenic NPs can induce ROS formation and activate angiogenesis factors (VEGF, fibroblast growth factor (FGF)) to accelerate wound healing. To clarify the exact mechanism of NP-mediated wound healing, extensive research on different phases of healing, toxicity, and biocompatibility is needed to enhance NPs’ therapeutic potential.

### 4.5. Osteoinductive and Angiogenetic Activities

Recently, scientists have been exploring various methods to enhance bone tissue regeneration. Important examples are metallic and metal oxide NPs. Materials for bone regeneration and remodeling should not only be biocompatible but also ought to exhibit anti-infective activity and promote angiogenesis. NPs have likewise appeared to have important antibacterial and antifungal properties. Moreover, it was found that they also have osteoinductive properties or the ability to induce bone formation after implantation. For example, Jadhav et al. [310] phyto-synthesized Au NPs by using *Salacia chinensis* to evaluate their osteoinductive activities. The NP exposure to GNPs and MG63 cell lines increased the percent of cell viability from 96 ± 3.7 (control) up to 138 ± 27.4, which confirmed their osteogenic potential. Consequently, it appears that such NPs may be used as active bone inductive material during implant placement. Plant (*Anogenissus latifolia*) polyphenol biosynthesized Au NPs that were found to be stable in a vast range of blood components were also utilized as an effective pain reliever and osteoinductive adjuvant of dental tissue implantation [311]. Similarly, ZnO NPs with an average size of 26 nm phyto-synthesized by *Scutellaria baicalensis* showed enhanced differentiation, proliferation, and mineralization during a 14-day period of cultivation with MG63 cells [312]. The authors proposed that the contact between the cells and biomaterial induced signal transduction pathway-regulated inductions, which influenced cell behavior. The biogenic ZnO NPs were non-toxic and biocompatible. Increasing the biocompatibility of the implant will ultimately lead to a longer life span of the graft and higher effectiveness of the implantation.

Angiogenesis is a vital process of the development of new blood vessels as a sequence of several cellular and molecular mechanisms [313]. This includes activation of endothelial cells in pro-angiogenic growth, degradation of capillary walls of the existing vessels, branch point formation inside the vessel, and migration of endothelial cells into the extracellular matrix and then to the starting point of angiogenetic stimulation [314]. The activation of endothelial cells can happen in response to specific conditions (such as hypoxia and inflammation) or during would healing to enhance tissue regeneration [315]. Angiogenesis is controlled by pro-angiogenic and anti-angiogenic factors such as vascular endothelial growth factor (VEGF), fibroblast growth factor (FGF), transforming growth factor- beta (TGF-β), tumor necrosis growth factor-alpha (TNF-α), IL-8, etc. [316]. The role of nanostructures in promoting angiogenesis is widely recognized in tissue engineering [317]. It was found that the application of magnetic stimulation can synergistically boost bone formation and pro-angiogenic potential more than just magnetic NPs alone [318]. For that reason, Moise et al. [319] bacterially (*Geobacter sulfurreducens*) synthesized Zn- and Co-doped magnetite NPs to tailor their magnetic properties. Negligible cytotoxicity of both moderately doped NPs on primary human bone marrow-derived mesenchymal stem cells (hMSCs) and human osteosarcoma-derived cells (MG63) was observed. A relatively strong AC susceptibility signal was detected from Zn-doped NPs compared to Co-doped NPs. With time the NPs were internalized by cells and stored within lysosomes. The osteogenic potential of hMSCs was unaffected by the uptake of Zn- and Co-doped NPs. It follows that utilizing biogenic NPs can yield superior bone regeneration accompanied by blood vessel formation in complex bone tissue engineering. However, more studies are required.

### 4.6. Anti-Viral Activity

Viruses are one of the contagious agents causing various diseases in humans. Some viral infections triggered by hepatitis, herpes-simples virus (HSV), influenza, COVID, human immunodeficiency virus (HIV), etc. cause pathologically complicated diseases and could be life-threatening. Available antiviral drugs, such as acyclovir, inhibit viral multiplication but have many side effects. Numerous drugs generated from plant and microbial sources have been recently formulated and commercialized as antiviral agents [320]. Moreover, metallic NPs are found to act as viricidal agents capable of blocking viral spread, interacting with viruses, and suppressing free virions [321]. The antiviral potential of NPs can be attributed to adsorption onto viral surfaces thus blocking the virus penetration and entry into the host [322], direct interaction of NPs with the viral proteins and viral genome [323], and inhibition of generic expression and production of viral constituents [324]. When supported by the viricidal potential of capping biocomponents, their anti-viral activity can be accelerated.

For instance, biosynthesized Ag NPs through *Andrographis paniculate*, *Phyllanthus niruri*, and *Tinospora cordifolia* demonstrated antiviral activity against *Chikungunya* virus [325]. The cytotoxicity assays in Vero cells revealed that *Andrographis paniculate* Ag NPs were the most toxic with a maximum non-toxic dose (MNTD) value of 31.25 μg/mL, followed by *Tinospora cordifolia* and *Phyllanthus niruri* Ag NPs. The cell viability tests also confirmed that Andrographis paniculate Ag NPs inhibited the virus to a maximum extent. Al-Sanea et al. synthesized Ag NPs by a biogenic route that were highly potent against SARS-CoV-2 [326]. The authors used strawberry (*Fragaria ananassa* Duch.) and ginger (*Zingiber officinale*) methanolic extracts for the biosynthesis of these Ag NPs with an average size of 5.9 and 5.8 nm, respectively, both with spherical shapes. The strawberry-synthesized NPs showed the highest antiviral activity against SARS-CoV-2 with IC_50_ value of 0.099 μg/mL. Molecular docking studies have shown that neohesperidin, a dereplicated compound, can bind to Aak1 (adoptor-associated kinase 1—a host kinase that regulates the intracellular viral trafficking during the entry) through hydrogen bonds and thus can act as a dual inhibitor for both Aak1 and viral NSP 16 (SAM-dependent methyltransferase that methylates the RNA cap). In another study, Ag NPs have been synthesized by a biogenic route through *Cinnnamomum cassia* bark extract and tested against H7N3 influenza A virus in Vero cells [327]. The biosynthesized NPs were found to be effective in both treatments when incubated with the virus before infection and when introduced after infection. The biomimetic NPs were also proved to be non-toxic to Cero cells up to 500 μg/mL. Biogenic Ag NPs (size of 12–28 nm) biosynthesized through an extract of mangrove *Rhizophora lalarckii* also showed anti-HIV activity by inhibiting HIV 1 type reverse transcriptase even at low doses [328]. In vitro, the mangrove fabricated Ag NPs exhibited an IC_50_ of 0.4 μg/mL on the HIV reverse transcriptase. Simultaneously, Dev et al. showed that Ag NPs conjugated with the anti-retroviral drug lamivudine were found to be highly prospective drug-delivery agents acting as a potent and selective inhibitors of type 1 and type 2 HIV [329].

Except for silver, gold NPs were also proven to be effective in antiviral therapy. Seaweed *Sargassium wightii* was used for the synthesis of biogenic Au and Ag NPs that were evaluated against herpes simplex virus (HSV-1 and HSV-2) [330]. The biogenic Au NPs showed a reduced by 70% cytopathic effect of HSV-1 and HSV-2 at 10 μL and 25 μL, respectively. Ag NPs showed a decrease in cytopathic effect by 70% of HSV-1 and HSV-2 at 2.5 μL. At the same time, the Au NPs were significantly non-toxic at all tested concentrations, while Ag NPs were toxic at higher concentrations.

Metal oxide NPs also demonstrated potential in viricidal therapy. For example, Yugandhar et al. synthesized biogenic CuO NPs by using fruit extract of *Syzygium alternifolium* [331]. The average size of the biogenic Cu NPs was 17.5 nm varying from 2 to 69 nm. The authors showed a significant growth inhibitory effect against Newcastle Disease Virus (NDV). The effective concentration of CuO NPs against NDV was 100 μg/mL.

It follows that by coupling the anti-viral activities of metallic NPs, biochemicals, and/or drugs, more accessible and low-cost anti-viral nanotherapeutics with minimal cytotoxicity can be produced.

### 4.7. Antiparasitic Activity

Parasites are organisms that live within other living species while utilizing their food and shelter. They are considered more pathogenic than bacteria to humans, since they can cause chronic diseases [332]. Parasitic infections are usually treated by chemotherapeutic agents and phytoextracts. However, some of the treatments are no longer effective because some protozoa have developed resistance [333]. Additionally, the bioavailability of antiparasitic drugs is low because of their short half-life and insolubility [334]. The synthesis of biogenic NPs is an approach to combine the efficacy of plant-based material with the achievements of biogenic nanotechnology to obtain effective antiparasitic therapeutics. For example, the parasiticidal effect of Karthik et al. reported a biosynthesis of Ag NPs from *Streptomyces* sp. and the obtained nanoparticles exhibited antiparasitic (acaricidal) activity against *Rhipicephalus microplus* and *Haemaphysalis bispinosa* [335]. Alajmi et al. observed antiparasitic activity of biosynthesized Ag NPs prepared by a combination of plant extracts of *Phoenix dactylifera* and *Ziziphus spina-chrisri* (traditionally used against toxoplasmosis) against oblige intracellular apicomplexan protozoan parasite *Toxoplasma gondii* [336]. As an alternative to standard sulfadiazine drug therapy, nanoparticle pretreatment prevented liver damage as determined by significant inhibition of hepatic NO levels and elevation in liver superoxide dismutase (SOD) and catalase (CAT) activities. NP treatment decreased proinflammatory cytokines and boosted the antioxidant enzyme activity of liver homogenate compared with an untreated control group. NP treatment induced a reduction in immunoreactivity to TGF-β and NF-kB in hepatic tissue, too. Improvements in the histological features of liver tissue and fewer degenerations were also observed.

Similarly, Leishmaniasis is a life-threatening disease caused by the parasite Leishmania. Some of the commercially applied anti-Leishmania drugs suffer from resistance mechanisms. For that reason, Ullah et al. synthesized Ag NPs through both chemical and biogenic methods from *Teucrium stocksianum* aqueous plant extract and evaluated their antileishmanial activity [337]. The study indicated strong antiparasitic activity against *Leishmania infantum* promastigotes for both NPs with higher efficacy for the biosynthesized (IC_50_ 30.71 ± 1.91 μg/mL) as opposed to the chemically obtained (IC_50_ 51.23 ± 2.2 μg/mL). At the same time, the MTT assay showed that the chemical Ag NPs exert higher toxicity than the biogenic Ag NPs. An efficient method of drug delivery to enhance the antileishmanial activity of miltefosine with Ag NPs was proposed by Kalangi et al. [338]. The biosynthesized Ag NP through *Anethum graveolens* leaf extract had an average size of 35 nm and demonstrated biocompatibility pertaining to >80% viability of macrophages. In combination with miltefosine (12.5 μM and 25 μM), the Ag NPs magnified the leishmanicidal effect of miltefosine by 2-folds. The enhanced effect of miltefosine (12.5 μM) in combination with AgNPs (50 μM) was confirmed by morphological aberrations and DNA fragmentations in promastigotes. Similarly, ZnO NPs synthesized by using *Aquilegia pubiflora* showed a dose-dependent cytotoxicity against *Leishmania tropica* (KWH23) with significant IC_50_ for both promastigote (48 μg/mL) and amastigote form (51 μg/mL) of the parasite [339]. In another study, antileishmanial properties of ZnO were enhanced by formulating bimetallic ZnO/Ag NPs that were synthesized using *Mirabilis jalapa* [340]. The activity of the bimetallic NPs was greater than monometallic ones while increasing with the increase of concentration. A possible mechanism of the antiparasitic activity of biosynthesized NPs can be their interaction with the cell membrane and cellular enzymes, causing intracellular damage and a high level of production of ROS that trigger apoptotic mechanisms in the cell.

## 5. Cytotoxicity of Biosynthesized NPs

Although nanomaterials and nanotechnology have developed considerably during the last 20 years, their potential toxicological effects on humans, animals, plants, and the environment have only currently received adequate attention. Previous studies discovered that NPs induce toxicity by a generation of ROS or increased oxidative stress in cells that can trigger cell death by inducing apoptosis or necrosis process [341]. When biogenic NPs are subject to drug-delivery application, they can suffer from instability in the hostile environment and bioaccumulation that can trigger biotoxicity. The biogenic techniques can impart substantial stability by capping with various agents [342]. According to Docea et al. the cellular uptake and colloidal stability of “coated NPs” are among the factors that control the toxicological features of NPs against cells [343]. However, the shape, size, and composition of NPs are characteristics that also determine biogenic nanomaterial toxicity [344]. Therefore, modulating these parameters will enable the designing of metallic NPs with improved interactions, biological activity at a target site, and low cytotoxicity. Sharma et al. [345] stated that the rate of NP dissolution can be controlled by the size of the material, capping, or surface functionalization. It was found that the cell viability (MTT analysis) of bone stem cells after incubation for 24, 73, and 96 h with biogenic and chemically synthesized Ag NPs was decreased but the toxicity of the biogenic NPs was substantially (11 folds) lower [223]. It is thought that the major toxicity of Ag NPs is associated with free silver ions that may negatively affect the eyes, skin, kidney, and liver cause respiratory problems and change blood cells [346]. Therefore, the non-toxic coatings (capping agents) provide a higher degree of biocompatibility and lower toxicity.

Rheder et al. observed that Ag NPs synthesized from an infusion of roots (AgNPR) and leaf extract (AgNPE) of the same plant (*Althea officinalis*) display different toxicity against A549 and V79 cell lines and none of them presented cytotoxicity towards HaCat cells [347]. The viability of the A549 and V79 cells presented cell death with low IC_50_ values when treated with AgNPE compared to AgNPR, but both materials exhibited physicochemical changes during exposure assays. In vitro, the cytotoxic effect of Ag NPs obtained by *Butea monosperma* bark extract against human PBMCs and leukemic KG-1A cell lines was tested by Pattanayak et al. [348]. In KG-1A the IC_50_ value of Ag NPs was 11.5 μg/mL while in human PBMCs it was 43.2 μg/mL. In leukemic cells, the ROS generation level increased over 4 folds at the effective dose and typical characteristics of apoptosis like plasma membrane blebbing and formation of apoptotic bodies were observed. Chromatin condensation and fragmentation also suggested that the Ag NPs deserved potential genotoxic effects. However, no in vivo studies have been performed.

Murugesan et al. [349] synthesized coated Ag NPs by curcumin derivate (ST06) and assessed their in vivo cytotoxicity in the Ehrlich Ascites carcinoma tumor-induced mouse model. ST06-Ag NPs with sizes ranging between 50 and 100 nm exhibited higher ani-tumor efficacy because of impaired angiogenesis than free ST06 or Ag NPs. In a dose of 5 mg/kg, the biogenic NPs showed no significant toxic effects in the animals. Using an acute inflammation model in Wistar rats, Moldovan et al. [350] demonstrated that the presence of some functional groups with biological origin on the NP surface can reduce cytotoxic effects. Spherical Ag NPs (10–50 nm) photosynthesized from aqueous extract of European cranberry bush (*Viburnum opulus* L.) decreased the level of cytokines in the soft hind foot pad tissue early after the induction of inflammation (2h) and reduced the increase in paw volume by inhibiting the inflammatory processes with edema formation.

It is thought that due to the higher concentration in blood circulation, Ag NPs are located in almost all vital organs, especially the liver [351]. The levels of antioxidant enzymes, such as CAT (catalase) and SOD (superoxide dismutase), decrease in the presence of tumors since cancer cells generate a large amount of hydrogen peroxide [352]. When treated with *F. religiosa* derived Ag NPs (5–35 nm) the levels of these liver antioxidant enzymes increased near to normal in Dalton’s ascite lymphoma mice models [353]. Histopathological studies also revealed recovery of liver architecture upon treatment with Ag NPs whereas hematological parameters revived to normal values after treatment. Similarly, *Morus alba* leaf extract formulated Ag NPs were examined for their hepatotoxicity in Wistar rats by intraperitoneal injection [223]. The authors observed that the therapy with Ag NPs at all doses (25, 50, and 100 μg/kg) significantly restored the metabolic enzyme activities, which indicated improved physiological functioning of hepatic tissue. Histopathological observation confirmed the healing of hepatic parenchyma and regeneration of hepatocytes.

However, in vivo, studies of biogenic Ag NPs synthesis using *Desmodium gangeticum* aqueous extract revealed obvious nephrotoxicity. This nephrotoxicity of both chemically and biosynthesized Ag NPs in a proximal epithelial cell line, renal mitochondria, and rats were evaluated by Vasanth and Kurian [354]. After 15 days of oral administration of Ag NPs (100 mg/kg) to the Wister rats, significant changes in renal architecture were seen in both receiving rats. The urine and blood chemistry data, as well as the renal epithelial cells and renal mitochondria, confirmed the toxic similarities between the Ag NPs produced by two different routes. Similarly, Ag NPs synthesized using sulfonated polysaccharide extract from *Sargassium siliquosum*, a brown alga, with polydisperse size (20–480 nm) [355], did not cause mortality to rats up to 2000 mg/kg BW but triggered elevation of serum creatinine and blood urea nitrogen. A low dose was effective in a revival of liver enzyme parameters to normal in intoxicated groups with paracetamol-induced liver injury. However, an apparent toxic effect on the kidney was also observed.

Many researchers have reported lower cytotoxicity of biomimetic Au NPs than Ag NPs irrespective of the cell type used [356]. For example, biosynthesized Au NPs from *Curcuma manga* were found to be cytocompatible with human lung fibroblast cells (MRC-5) and human colon fibroblast cells (CCD-18Co) [357]. These Au NPs also demonstrated hemocompatibility with less than 10% of hemolysis without any aggregation of erythrocytes. At the same time, Nandhini et al. reported the biogenesis of gold NPs from *Enterococcus* sp. RMAA and studied its cytotoxic potential against human hepatocellular cancer (HCC) cell lines. It was revealed that Au NPs resulted in about 20% cytotoxicity of cancer cell lines due to oxidative stress and reduced mitochondrial membrane potential that eventually induces cytochrome c release into cytosol leading to apoptosis of cancer cells [358]. Moreover, biosynthesized Au NPs using plant extract were shown to be non-toxic to normal L-cells at various concentrations (1 to 100 μL) [359]. The results underlined the potential of an extract-based method for the synthesis of Au NPs for therapy and anticancer treatment.

In another study, in vitro toxicity of pullulan-supported Au NPs loaded with 5-fluorouracil and folic acid against HepG2 cells was studied [360]. It was concluded that the amount of 5-fluorouracil required to achieve 50% of growth inhibition was much lower for the conjugate than in free 5-fluorouracil. Additionally, the in vivo distribution of gold was increased mostly in the liver than in other organs. Lee et al. reported the synthesis of chitosan-capped gold nanospheres and nanostars, derived from green tea extracts. At the same time, gold nanorods were prepared by a conventional method. The authors demonstrated cytotoxicity of the biogenic Au NPs towards four cancer cell lines, of which the highest toxicity towards hepatocyte carcinoma was exhibited in nanorods, followed by nanostars and finally nanospheres. Further, the cellular uptake of Au NPs by human hepatocyte carcinoma cells (HepG2) followed the order nanospheres > nanorods > nanostars [361]. Moreover, the study of Mironova et al. demonstrated that after 3 days of incubation, 142 μg/mL of 13 nm AuNPs cause nearly 40% apoptosis in human fibroblast cell lines while only 13 μg/mL of 45 nm AuNPs triggered the same effect [362]. However, after 14 days full recovery occurred for the fibroblast cells after the removal of both particles. Hence, it is demonstrated that the shape, size, and origin of metal NPs play a crucial role in therapy and as drug delivery agents.

Eisa et al. reported the biosynthesis of TiO_2_ NPs from titanium isopropoxide and lupin bean extracts and demonstrated its antimicrobial and cytotoxic activities. These biogenic TiO_2_ NPs exhibited cytotoxicity against breast cancer cell (MCF-7) lines with an IC_50_ of about 41.1 µg. The authors compared the cytotoxicity of biogenic TiO_2_ NPs with Vinblastine against breast cancer cells, where the former showed greater efficacy in cancer therapy [363]. Selim et al. reported cytotoxic effects of a plant (*Deverra tortuosa* aqueous extract)-derived ZnO NPs with an average size of 15.2 nm on cancer cells. The authors demonstrated anticancer activity in vitro model against human colon (Caco-2) and lung (A549) cancer cell lines and compared them to human lung fibroblast (WI38) cell lines using MTT assay [364]. The biogenic NPs showed remarkable selective cytotoxicity against both examined cancer cell lines. As of now, one of the most challenging fields in anticancer treatment is the development of new anticancer drugs with minimal side effects and enhanced efficacy and selectivity, and the biosynthesis of NPs could pave the way toward more precise and effective anti-cancer treatment. However, it follows that the method of biosynthesis, reducing and capping biological material, type of NP, and its shape are all determinative for the toxicity of that NP.

## 6. Future Perspectives and Outlook

Nanobiotechnology, by combining chemistry, engineering, biology, and medicine, offers various benefits in treating severe or chronic human diseases via target-oriented delivery. The biosynthesis of NPs is now an important branch in nanobiotechnology. By combining the unique properties of metallic NPs with the benefits of conjugated synthesis and surface functionalization, a wide range of opportunities give room for discoveries and various biomedical applications. From the relevant studies, it appears that biogenesis is not only an eco-friendly and inexpensive alternative for conventional NPs production but also an option to design multifunctional nanomaterial that can effectively treat diseases or deliver drugs without disturbing the organism or causing less harmful effects. Biogenic synthesis usually allows synergy between the nanoparticle and the substance from the organism employed for production. The use of genetically modified microorganisms for the biogenic production of NPs with a desired shape and size is a novel and promising approach in this area. The potential activity and cytotoxicity of these biosynthesized NPs will be affected by the presence and type of coating on their surface.

However, most of the biosynthesis methods are still under development, and issues such as purification and separation of NPs are to be further overcome. Moreover, there are certain gaps in knowledge in a thorough understanding of mechanisms and biochemical pathways of bio-mediated synthesis of NPs. When dedicated to biomedical purposes, it is vital to know the active moieties that bind and participate in synthesis and provide surface stability and biocompatibility. Large-scale production of biocompatible nanostructures with narrow size distribution is another challenge in front of recent biotechnology methods. The heterogeneity may hamper their use in different biomedical applications where precise shape and size are of vital importance. Exact determination of the reducing components should be performed to establish precise and repeatable methods for biosynthesis. Moreover, the structure and composition of biogenic NPs together with their coatings must be carefully analyzed and the potential interactions with the biological system must be evaluated. The possibility of aggregation and formation of agglomerates that can affect the properties and bioavailability of the individual NPs should also be assessed to avoid unexpected toxic effects. Actinomycetes and plant-mediated metal nanoparticle synthesis have shown promise as therapeutic agents whose mechanism of action is yet to be explored.

Concerning the biomedical application of these NPs, another issue is related to their distribution profile, release kinetics, and clearance in the organism. However, most of the developed conjugates by biogenic processes are biomaterials with reduced toxicity when compared to their physiochemically synthesized counterparts. As such systems, biogenic NPs are advantageous to avoid the main issue faced by modern drug-delivery systems: nano-cytotoxicity. The fabrication of biogenic nanocomposites for drug delivery in a more intelligent and focused approach, it seems, will have a bright future in biomedicine.

## Figures and Tables

**Table 1:** Plant-derived biosynthesized metallic NPs.

Plant	Source	NPs	Size, nm	Morphology	Application/ Tested Objects	Ref
*Cucumis anguria*	Leaf	Ag	11–27	Spherical	Antibacterial against *S. aureus*, *E. coli*	[104]
*Madhuca longifolia*	Flower	Ag	30–50	Spherical and oval	Antibacterial against *E. coli*, *S. typhimurium*, *B. cereus*, *S. saprophyticus*	[28]
Banana waste	Peduncle	Ag	14.1	Spherical	Antimicrobial against *S. aureus*, *E. coli*, *C. albicans*, *A. niger*	[105]
*Trachyspermum ammi*	Seed	Au	16.6	Spheroidal	Anti-biofilm against *L. monocytogenes* and *S. marcescens*; anticancer against HepG2 cell lines	[106]
*Garcinia mangostana*	Pericarp	Au, Ag	13.7 ± 5.1 31.1 ± 4	Nanodumbbell shapes	Anticancer, antioxidant against colon cancer Col320 cell line	[107]
*Asparagus racemosus*	Root	Ag, Au, Ag-Au	10–50	Spherical	Antibacterial against *P. aeurgnosia* and *S. aureus*, anti-inflammatory in NK92 cells	[108]
*Rhanterium epapposum*	Flower	Ag Au Zn	16.1 17.9 23.5	Spherical, triangular, hexagonal	Antifungal against *C. albicans* and *A. melleus*, cytotoxic analysis against breast adenocarcinoma MCF-7, hepatocellular carcinoma HepG-2 and colorectal carcinoma HCT 116 human cell lines	[109]
*Rosmarinus officinalis*	Leaf	Pd	15–90	Semi-spherical	Antibacterial against *E. coli*, *S. aureus*, *S. epidermidis* and *M. lutens*; antifungal against *C. albicans*, *C. parapsilolis*, *C. glabrata* and *C. krusei*	[110]
*Allium fistulosum*, *Tabernaemontanadivaricata*, and *Basella alba*	Leaf	Pd	20–50	Spherical	Antibacterial against *Basella alba*, *Allium fistulosum*; antifungal against *C. albicans*, *A. flavus*, and *Penicillinium* sp.; antioxidant (free radical scavenging), antidiabetic (α-amylase inhibition)	[111]
Saudi’s dates	Fruit	Pt	1.3–6.6	Spherical	Anticancer against colon cancer HCT-116, breast cancer MCF-7 and hepatocellular carcinoma HePG-2 cell line	[112]
*Celastrus paniculatus* Willd.	Leaf	Cu	2–10	Spherical	Antifungal against *F. oxysporum*	[113]
*Cissus vitiginea*	Leaf	Cu	2–20	Spherical	Antioxidant, antibacterial against *E. coli*, *Enterococcus* sp., *Proteus* sp. *Klebsiella* sp.	[114]
*Syzygium aromaticum*	Bud	Cu	12–15	Spherical	Antimicrobial against *Staphylococcus* spp., *Bacillus* spp., *Pseudomonas* spp., *E. coli*, *A. niger*, *A. flavus* and *Penicillium* spp.	[56]
*Azadirachta indica*	Leaf	CuO	NA	Spherical	Anticancer against breast cancer MCF-7 and Hela cell lines	[115]
*Achillea millefolium*	Leaf	CuO	28	Semi-spherical	Antibacterial against *S. aureus*, *M. tuberculosis*, *E. coli*, *K. pneumoniae*, *P. mirabili*, *C. diphtheriae*, *S. pyogenens*; antifungal against *C. albicans*, *A. flavus*, *M. canis* and *G. glabrata*	[116]
*Musa ornate*	Flower	Fe	43.7	NA	Antibacterial against *S. aureus*, *S. agalactiae*, *S. enterica*, *E. coli*	[117]
*Ficus carica*	Leaf	Fe_3_O_4_	43–57	Multiform	Antioxidant (DPPH free radical scavenging)	[118]
*Platanus orientalis*	Leaf	Iron oxide	30–40	Spherical	Antifungal against *A. niger* and *M. piriformis*	[119]
*Mentha pulegium* (L.)	Leaf	ZnO	38–49	Semi-spherical	Antimicrobial against *E. coli* and *S. aureus*	[68]
*Conyza canadensis*	Leaf	ZnO	-	Spherical	Antibacterial against *E. coli* and *S. aureus*	[120]
*Costus pictus* D. Don	Leaf	ZnO	40	Elongated, hexagonal, and rod shape	Antimicrobial against *S. aureus*, *B. subtilis*, *E. coli*, *S. parathyphi*, *A. niger*, *C. albicans* anticancer against Dalton’s ascites cells	[121]
*Albizia lebbeck*	Stem bark	ZnO	66.6	Irregular spherical	Antimicrobial against *B. cereus*, *S. aureus*, *E. coli*, *K. pneumoniae* and *S. typhi*, antioxidant (hydrogen peroxide free radical scavenging), anticancer against breast cancer MDA-MB 231 and MCF-7 cell lines	[122]
*Ailanthus altissima*	Plant extract	ZnO	13.3	Spherical	Antibacterial against *S. aureus*, *K. pneumonia*, *E. coli*, *S. pyogenes*; antioxidant (DPPH free radical scavenging)	[123]
*Punica granatum*	Peel	ZnO	118.6	Irregular nanorods	Antibacterial against *S. aureus*, *E. aerogenes*, *P. aeruginosa*, and *K. pneumoniae*	[124]
*Trigonella foenum-graecum*	Leaf	MgO	13	Spherical	Antibacterial against *E. coli, Bacillus* spp., and *S. aureus*	[125]
*Ceratonia siliqua*	Leaf	CeO_2_	22	NA	Antioxidant (DPPH free radical scavenging), cytotoxic against breast cancer MCF-7 cell lines	[126]

**Table 2:** An overview of different nano-vector microorganisms used for the production of metallic NPs employed in biomedical applications.

Class Organism	Metallic NPs	Intra/Extracellular Synthesis	Species	Morphology	Size, nm	Application/ Tested Objects	Ref.
Bacteria	Au	Extracellular	*Paracoccus haeundaensis*	spherical	20.9 ± 3.5	Antioxidant (DPPH free radical scavenging), cytotoxicity against normal kidney cells HEK 293, anticancer against lung carcinoma A549 and gastric adenocarcinoma AGS cell lines	[190]
	Au	Intracellular	*Lactobacillus kimchikus*	spherical	5–30	Antioxidant (DPPH free radical scavenging)	[191]
	Ag	Intracellular	Transformed *E. coli*	spherical	20	NA	[192]
	Ag	Extracellular	*Bacillus brevis*	spherical	41–68	Antibacterial against *S. aureus* and *S. typhi*	[193]
	Ag	Extracellular	*Bacillus subtilis*	spherical	3–20	Antimicrobial against *S. aureus*, *S. epidermidis*, *K. pneumoniae*, *E. coli* and *C. albicans*	[29]
	Pt	Intracellular	*Acinetobacter calcoaceticus*	cubic	2–3	NA	[194]
	Cu	Intercellular	*Streptomyces capillispiralis* Ca-1	spherical	3.6–59	Antimicrobial against *S. aureus*, *B. subtilis*, *B. dimenuta*, *P. aeruginosa*, *E. coli*; Antifungal against *C. albicans* and *A. brasiliensis*	[195]
	ZnO	Extracellular	*Pseudomonas putida*	spherical	44.5	Anti-biofilm against *B. cereus* and *E. faecalis*, antibacterial against *P. otitidis*, *P. oleovorans*, *A. baumannii*, *B. cereus*, *E. faecalis*	[196]
	ZnO	Extracellular	*Staphilococcus aureus*	irregular	10–50	Antibacterial against *E. coli*, *S. aureus*, *S. epidermis*, *E. faecalis*, *K. pneumonia*, *P. aeruginosa*	[69]
	ZnO	Intercellular	*Serratia nematodipila*	spherical	15–30	Antibacterial against *X. oryzae* and antifungal against *Alternaria* sp.	[197]
	TiO_2_ ZnO	Extracellular	*Halomonas elongata*	spherical	104.6 18.1	Antibacterial against *E. coli* and *S. aureus*	[198]
	TiO_2_	Extracellular	*Streptomyces* sp.	spherical	30–70	Antimicrobial activity against *E. coli*, *S. aureus*, *C. albicans*, *A. niger*; antibiofilm against *P. aeruginosa*	[199]
	CuO	Extracellular	*Streptomyces* sp.	spherical	78–80	Antifungal against *F. oxysporum*, *P. ultimum*, *A. niger*, *A. alternata* cytotoxicity against Vero and Caco-2 cell lines	[200]
	CuO	Extracellular	*Streptomyces* sp.	spherical	1.7–13.5	Antibacterial against *E. faecalis*, *S. typhimurium*, *P. aeruginosa*, *E. coli*; Antifungal against *C. albicans*, *R. solani*, *A. niger*	[201]
	Iron oxide	Extracellular	*Proteus vulgaris* ATCC-29905	spherical	19.2–30.5	Antibacterial against methicillin resistant *S. aureus*; anticancer against U87MG-glioblastoma cancer and HT-29 cancer cell line	[202]
Fungi and Yeast	Au	Extracellular	*Cladosporium cladosporioides*	quasi-spherical	60	Antioxidant (DPPH and FRAP assay), antimicrobial against *S. aureus*, *B. subtilis*, *P. aeruninosa*, *A. niger*	[203]
	Ag	Extracellular	*Cladosporium cladosporioides*	spherical	30–60	Antimicrobial against *S. aureus*, *B. subtilis*, S. *E. coli*, *S. epidermidis*, and *C. albicans*; antioxidant (DPPH assay)	[204]
	Ag	Extracellular	*Beauveria bassiana*	triangular, circular, hexagonal	10–50	Antibacterial against *E. coli*, *P. aeruginosa*, *S. aureus*	[30]
	Pt	Extracellular	*Fusarium oxysporum*	spherical	25	Antibacterial against *E. coli* Antioxidant (DPPH assay)	[205]
	Pd	Intracellular	*Agaricus bisporus*	triangular spherical	13–18	Antibacterial against *S. aureus*, *S. pyrogens*, *B. subtilis*, *E. aerogenes*, *K. pneomoniae*, *P. vulgaris*, anticancer against PK13 cell lines, anti-inflammatory with RBC cells Antioxidant (DPPH method)	[206]
	Cu	Extracellular	*Aspergillus niger*	round	500–800	Antidiabetic (α-glucosidase assay), anticancer against human hepatocellular carcinoma Huh-7 cell lines; antibacterial against *E. coli*, *S. aureus*, *K. pneumoniae*, *M. luteus*, *B. subtilis*	[207]
	ZnO	Extracellular	*Aspergillus niger*	spherical	35	Antibacterial against *E. coli*; cytotoxicity against breast cancer MCF-7 cell lines	[208]
	ZnO	Extracellular	*Xylaria acuta*	hexagonal	34–55	Antimicrobial against *S. aureus*, *B. subtilis*, *P. aeruginosa*, *E. coli*	[70]
	ZnO	Extracellular	*Aspergillus niger* and *F. keratoplasticum*	nanorods hexagonal	8–32 10–42	Antimicrobial against *B. subtilis*, *S. aureus*, *P. aeruginosa*, *E. coli*	[209]
	CuO ZnO	Extracellular	*Penicillium chryogenum*	spherical hexagonal	10.5–59.7 9–35	Antibiofilm against *S. aureus* and *P. aeruginosa* Antimicrobial against *S. aureus*, *B. subtilis*, *P. aeruginosa*, *E. coli*, *S. typhimurium* and fungi *F. solani*, *F. oxysporum*, *S. sclerotia*, *A. terreus*	[210]
	TiO_2_	Extracellular	*Pleurotus djamor*	spherical	31	Anticancer against A549 cell lines, Antibacterial against *P. fluorescens*, *S. aureus*, *C. diphtheriae*	[211]
Algae	Ag	Intracellular	*Padina* sp.	spherical	25–60	Antimicrobial against *S. aureus*, *B. subtilis*, *E. coli*, *S. typhi*, *P. aeruginosa*	[212]
	Ag	Extracellular	*Gelidium corneum*	spherical	20–50	Anti-biofilm and antibacterial against *C. albicans* and *E. coli*	[213]
	Au	Extracellular	*Cystosuira baccata*	spherical	8.4	Anticancer against colon cancer cell lines HT-29 and Caco-2	[214]
	Pd	Extracellular	*Spirulina patensis*	spherical	10–20	-	[215]
	Pd	Extracellular	*Chlorella vulgaris*	spherical	5–20	-	[216]
	Pt	Extracellular	*Padina gymnospora*	spherical, octahedral	10–60	Antimicrobial against *E. coli*, *L. lactis*, *K. pneumoniae* with no hemolytic activity in vitro	[217]
	Cu	Extracellular	*Botryococcus braunii*	cubic, spherical, triangular	10–70	Anti-microbial against *P. aeruginosa*, *E. coli*, *K. pneumoniae*, *S. aureus* and *F. oxysporum*	[218]
	Fe_3_O_4_	Extracellular	*Colpomenia sinuosa* and *Pterocladia capillacea*	nanospheres	11.2–33.7 16.9–22.5	Antibacterial against *E. coli*, *P. aeruginosa*, *S. typhi*, *V. cholera*, *B. subtilis*, *S. aureus* Antifungal against *A. flavus* and *F. oxysporum*	[219]
	ZnO	Extracellular	*Sargassium muticum*	hexagonal	30–57	Anti-angiogenetic and apoptotic effect on human liver cancer cell line HepG2	[71]
	ZnO	Extracellular	*Arthrospira platensis*	spherical	30–55	Antimicrobial against *B. subtilis*, *S. aureus*, *P. aeruginosa*, *E. coli* and *C. albicans*	[220]
Viruses	Au	intracellular	*Tobacco mosaic virus* (TMV)	spherical	5	-	[221]
	Au	intracellular	Genetically modified *Tobacco mosaic virus* (TMV)	spherical	9–33	-	[160]
	Au Ag	intracellular	Plant pathogenic virus of *Squash leaf curl China virus*	spherical	5–12 5–20	Cytotoxicity against A549 lung cancer cell line	[162]
	Pd Pt Au	Intra- and extracellular	*Tobacco mosaic virus* (TMV)	spherical	3.4 ± 0.5 3.1 ± 0.5 2.9 ± 0.5	Catalytic (allyl alcohol hydrogenation)	[222]

**Table 3:** Biosynthesized NPs from different species with anticancer activity, their size, shape, drug conjugation and the main outcome of the research.

NPs	Source	Size, nm	Shape	Drug	Tested Object	Main Outcome	Ref.
Ag	*Abelmoschus esculentus* (L.)	21.3	Spherical	-	Jurkat cells (human T-cell lymphoma)	-Antiproliferative effect in a dose-dependent manner with an IC50 value of 16.15 μg/mL -Triggered high levels of ROS; -Loss of integrity of mitochondrial membrane;	[253]
Ag	*Punica granatul* leaf extract	41.7–69.6	spherical	-	Cervical cancer HeLa cell line	-IC_50_ for inhibiting 50% of the Hela cell line was 100 μg/mL; -at a concentration of 100 μg/mL Ag NPs induced apoptosis by fragmentation of DNA	[254]
Ag	*Punica granulatum* leaf extract	35–60	Spherical	-	Liver cancer cell line HepG2	-IC_50_ for the HepG2 cell line was 70 μg/mL; -In vitro free radical scavenging activity.	[255]
Ag	*Taraxacum officinale*	5–30	Spherical	-	Liver cancer cell line HepG2	-High cytotoxic effect against HepG2	[256]
Ag	*Olax scandens* leaf extract	20–60	Spherical	-	B16 mouse melanoma cell line; A549 human lung cancer cell line; MCF7 human breast cancer cells	-Inhibition of proliferation of both A549 and B16 in a dose-dependent manner; -Compared to chemically synthesized Ag NPs, the biogenic showed biocompatible nature with normal cells;	[257]
Au	*Peltophorumpterocarpum*	65–149	Spherical	Dox	Lung (A549) and melanoma (B16F10) cancer cell line, C57BL6/J mice	-Nano-conjugate cellular uptake and drug release were faster than pure Dox; -No significant changes in hematology, serum clinical biochemistry, or histopathology in C57BL6/J mice were observed after 7 days	[258]
Ag	*Bacillus licheniformis*	20–80	Triangular	5-aminoevulinic acid (ALA)	Skin melanoma (B16F10), epidermoid carcinoma (A431)	-Higher cytotoxicity on both cell lines than the pure ALA and Ag NPs;	[259]
Au	*Syzygium aromaticum*	12–20	Spherical	-	Human lymphoma SUDHL-4 cell line	-The IC_50_ of Au NPs after 48 h was 30 μM; -Au NPs induced ROS accumulation and dose and time-dependent manner; -Apoptosis mechanisms involved caspase activation, formation of pores in the mitochondrial membrane, and release of cytochrome c; -The NPs decrease the growth and increase apoptosis of cells	[260]
Ag (Starch coated)	Meadow grass (*Poa anua*)	36.7 ± 7.9	Spherical	*Euphorbia dracunculoides* Lam. Plant extract	Human embryonic kidney (HEK293), Hep3B liver cancer, SCC-7 murine cancer cell line, Sprague Dawley rats	-Up to 96% drug loading efficacy was observed; -Good biocompatibility (90–100%) with HE293 at high concentrations (up to 1000 μg/mL) -at concentration 400 μg/mL of Hep3B and SCC-7 decreased to 20 and 30%, respectively after 72 h; -IC_50_ of Au NPs was 113.3 μg/mL; -Significantly low cytotoxicity in vivo of the bio-drug NP conjugates	[261]
Au	*Elettaria cardamomum* seeds	15.2	Spherical	-	Cervical cancer (HeLa) cell line	-the IC_50_ of Au NPs was 42.6 μL; -Penetration of Au NPs through the cell membrane; -Shrinking, rounding granulation morphological changes	[262]
Au	*Vitex negundo* (reducing agents) and Arabic gum (capping agent)	98.7 ± 1.9	Spherical	Epirubicin	Lung adenocarcinoma A549 cell line	-The amount of epirubicin bound was 85%; -The IC_50_ for value NP conjugate was around 4 μg/mL whereas for the drug alone was 24 μg/mL -The drug release from the conjugate was slow, thus the cytotoxicity and therapeutic efficacy were higher	[263]
Au	*Paracoccus haeundaensis* BC74171^I^ bacterium	20.9 ± 3.5	Spherical	-	A549 and AGS cancer cell lines	-Au NPs exert an antiproliferative effect on cancer cells and did not affect the normal cells HEK293 and HaCaT up to a concentration of 200 μg/mL; -The percentage of cell growth inhibition of AGS cells was higher than that of A549 indicating selective cytotoxicity;	[190]
Au	*Magnifera indica*	55.5–65.5	Spherical	-	MDA-MB-231 and breast cancer mice	-Highly effective in controlling the growth of breast cancer in a dose-dependent fashion;	[264]
Au	Peptides	100–150	Spherical	Dox	Hela and nude mice	-Tumor inhibition in nude mice via tumor and tail vain was 66.7 and 57.7%, respectively; -Efficient Dox transport vector for cervical cancer therapy.	[265]
Au	*Cyclopia intemedia* and magniferin	20	Spherical and Triangular	Dox	Human colon cancer (Caco-2), prostate cancer (PC-3) and glioblastoma (U87) cell lines; normal breast epithelial cell lines	-In combination with Dox, antitumor effects are augmented. -At the same time, relatively low cytotoxicity to normal cells was observed	[266]
Au/Pt	*Phragmites* *australis* leaves	35.1 ± 2.7	Flower-like shape	Dox	Breast (MCF-7) and lung (A459) cancer cell line	-pH-controlled dependency response under acidic tumor conditions; -A three-fold cell death to cancer cells compared to Dox alone; -Dox-conjugated Au/Pt NPs exhibited time-release phosphatidylserine exposure; -Cell-specific response against MCF-7 compared to A459	[267]
Fe_2_O_3_	*Psoralea corylifolia*	39	Spherical	-	Renal tumor MDCK and Caki-2 cell lines	-Strong cancer cell growth inhibiting in a dose-dependent manner	[268]
Fe_2_O_3_	*Mentha piperita* leaves	17.9	Polygonal (rhombic and hexagonal)	Dox	Breast MCF-7 cancer cell line	-The IC_50_ of Fe_2_O_3_ NPs against cancer cells was 0.5 μg/mL while at 1 μg/mL their toxicity to normal was negligible; -71% Dox loading efficacy; -NPs-Dox conjugates significantly inhibited the tested cancer cells compared to free drug;	[269]
Fe_2_O_3_	*Rhus punjabensis* extract	41.5 ± 5	Rhombohedral		HL-60 leukemia and DU-145 prostate cancer cell line	-The NPs were active against both cancer cells and leaved health control cell unaffected;	[270]
Fe	*Streptomyces* sp.	65–87	Spherical		DU145 and prostate cancer (PC3) cell line	-The IC_50_ for value NPs was around 65 μg/mL; -The color staining revealed an antiproliferative effect of the NPs on tumor cells;	[271]
CuO	Bean extract	26.6	Spherical, hexagonal, uneven shape	-	Human cervical carcinoma (HeLa) cell line	-The IC_50_ for value NPs was around 13 μg/mL increased ROS and lipid peroxidation of liposomal membrane; -Alteration in the mitochondrial structure; -Inability of cells to proliferate.	[272]
ZnO	*Leucaena leucocephala*	50–200	Hexagonal	-	Breast cancer (MCF-7) and prostate cancer (PC3) cell line; Dalton lymphoma ascites (DLA) cell line	-The IC_50_ for value NPs was around 103 μg/mL -Better cytotoxic activity on PC-3 than MCF-7 cell line; -Against DLA cells the biosynthesized ZnO NPs revealed 92% inhibition with a concentration of 200 μg/mL;	[273]
TiO_2_	*Cinnamomum tamala*	23	Irregular	-	Human prostate cancer (D145) cell line	-TiO_2_ NPs showed a dose-dependent anticancer effect	[274]
Au/ CuO/ ZnO	*Verbena officinalis* L. extract	35	Spherical	-	Jurkat cell line	-The IC_50_ for value NPs was around 0.64 μmol; -Within 24 h over 80% of the cells cultured in the presence of Au/CuO/ZnO NPs at a concentration of 10 μmol exhibited sighs of late apoptosis; -about 60% of the cells at a concentration of 100 μmol underwent necrosis.	[275]

## Data Availability

Not applicable.
